# A practical guide to placental examination for forensic pathologists

**DOI:** 10.1007/s12024-019-00214-2

**Published:** 2019-12-24

**Authors:** Namita Mittal, Roger W. Byard, Jane E. Dahlstrom

**Affiliations:** 1 Anatomical Pathology, ACT Pathology, The Canberra Hospital, Canberra Health Services PO Box 11, Woden, ACT 2606 Australia; 2 Faculty of Medicine, University of Adelaide, Frome Rd., Adelaide, 5005 Australia; 3 Forensic Science SA, GPO Box 2790, Adelaide, 5001 Australia; 4 College of Health and Medicine, Australian National University Medical School, Canberra, ACT 2601 Australia

**Keywords:** Placenta, Neonate, Maternal, Fetal, Forensic, Sudden death, Stillbirth

## Abstract

The placenta is a complex interface organ that may hold clues to the reasons for fetal, neonatal or maternal demise. For this reason, placental examination should be a mandatory part of all perinatal or maternal autopsies. While published protocols for the examination of the placenta exist, they are often not adopted. The following review provides practical guidelines for placental examination, with discussion of specific medical conditions that can negatively impact upon the fetus, neonate or mother involving placental pathology to cause death. The review aims to discuss concepts, with illustrations, that forensic pathologists may not routinely focus on in death investigations that may either contribute or mask the cause of a fetal or neonatal death, or are associated with a recurrence risk. While it is recognized that many forensic facilities do not have formal guidelines for placental examination, involvement of local perinatal pathology services in cases is one way of obtaining additional specialist expertise.

## Introduction

The placenta, in representing the interface between the fetus and maternal host, acts as a macro-membrane between the two circulations: fetal-placental and maternal-placental. Due to its villous and vascular structures, the placenta facilitates the development of breathing, endocrine secretion, metabolic changes and immune processes of the fetus. Dysfunction of the placenta can therefore result in perinatal, or less commonly, maternal death [1–7].

In order to derive clinically useful information from the placenta, meticulous macroscopic and microscopic examination is needed, in the context of the clinical circumstances [8]. As with many organs, the placenta can only react in a limited number of ways to a wide variety of insults. Frequently more than one placental pathology may be present to contribute directly to an adverse outcome. The present article reviews indications for placental evaluation, the approach to placental assessment and key findings associated with diagnoses that may give rise to perinatal death.

## Benefits of placenta examination

Issues of causation in relation to placental pathology are complex and often multifactorial [9]. The main benefits of placental examination include:

1. Provision of an audit of antenatal clinical judgment and management;
2. Identification of etiologies and pathological processes contributing to or causing an adverse pregnancy outcome;
3. Improved management of subsequent pregnancies by the identification of conditions known to have recurrence risks or which may be either treatable or preventable;
4. Assessment of factors contributing to poor outcomes as a factual basis for resolving medicolegal issues.

Pathological findings in the placenta may be helpful in understanding fetal, neonatal or maternal deaths in one of two ways [9]. Firstly, the placenta itself may be abnormal and thus contribute directly to the adverse outcome. Primary lesions of the placenta, such as massive perivillous fibrinoid deposition, chronic histiocytic intervillositis of unknown etiology, high grade chronic villitis of unknown etiology, decidual arteriopathy leading to placental ischemia and/or infarction and large chorangiomas, fall into this category. Secondly, the placenta may harbor abnormalities that indicate the presence of an adverse intrauterine environment. An example is the presence of a sizeable intervillous hemorrhage within a placenta that is markedly pale and hydropic, and in which villous capillaries show the presence of nucleated red blood cells, indicating the presence of significant fetomaternal hemorrhage [9].

The gross and microscopic features of the placenta change over gestation and so understanding the normal anatomy and histology of the placenta is essential. This is well reviewed by Avagliano et al. [10].

## Placental examination: Step wise guide

### Clinical history

The clinical history is crucial in placental assessment. For example, the gestational age of the fetus/neonate is important as the placental disc weight increases over gestation, and placental villi mature over time. Thus, while the placental weight or villous histology may be normal for a term gestation, they could be quite abnormal in a premature fetus/neonate or vice versa. Equally the fetoplacental weight ratio is an indicator of adequacy of placental reserve capacity and this will change over gestation [11]. Table 1 highlights key aspects of the clinical history that should be obtained prior to placental assessment. Use of published templates for documenting the clinical history can assist in obtaining standardized information [12]. Alternatively, the birth record can be requested if such information is contained therein. Details on death investigation outside of the hospital setting has been previously reviewed [13]. In such cases the maternal/obstetric history may not be available. If the placenta is found without a fetus the gestational age can be estimated from the combination of placental weight and dimensions compared to published normative data, and the histological maturation of villi (which is known to evolve with gestational age). If fetal remains are present, then radiology of the fetal bones can provide an estimate of fetal age and thus the expected weight and placental histology.

**Table 1:** Key clinical information

Gestational age	
Birth weight	
Gender of infant	
Apgar scores	
Maternal complications of pregnancy	
Fetal complications of pregnancy	
Labour	
Route of delivery	
Total umbilical cord length	

### Placental handling and transport

The placenta should be sent fresh for pathological assessment. This enables three key ancillary tests to be performed. The first is microbiology. This should be routinely performed as infection remains an important cause of perinatal mortality for both the neonate and mother. A practical guide to performing these tests can be found on the Royal College of Pathologists of Australasia (RCPA) website [14]. Swabs should be taken between the amnion and chorion; as well as taking of a 10mm^3^ sample of placental disc tissue for tissue bacterial culture, and if appropriate, viral assessment (usually via molecular testing such as polymerase chain reaction [PCR]). It is useful to take an additional 10mm^3^ sample of placenta that then can be stored at -70 °C for metabolic studies if this is felt to be appropriate. Thirdly, fresh tissue (umbilical cord or placental disc) should be sent to cytogenetic analysis to enable formal cytogenetic study or molecular karyotyping. If the placental disc is sampled for karyotyping it is important to not include decidua as this will result in maternal contamination of the specimen. If samples are collected for identification purposes, for example in the absence of an infant or fetal parts, or for identification of the mother fresh samples should be taken from the placenta disc or umbilical cord (to provide fetal DNA) and the decidua (to provide maternal DNA). Sterile techniques should always be used. If a delay is anticipated between delivery and receipt of the placenta, or its examination, refrigeration at 4 °C is an alternative to fixation []. It is recognized that these facilities may not be available in standard forensic morgues and so liaison with the local perinatal autopsy service may be advisable.

The placenta should ideally be assessed in both the fresh and fixed state as lesions such as early infarcts and fetal vascular malperfusion can be more readily identified in the fixed state.

### Gross pathological examination and sampling

Many placental lesions are diagnosed solely by gross examination, and the extent of the pathologic processes is best recognized by assessment of the whole specimen [16]. Protocols and techniques for placental examination and sampling are readily available [14]. The Amsterdam Placental Workshop guidelines are recommended [17] and a check list is provided in Table 2.

**Table 2:** Macroscopic assessment and sampling

Placental weight	
Placental disc measurement (3 dimensions)	
Placental disc complete?, shape, colour, chorionic vessels, abnormalities	
Umbilical cord insertion (mm from nearest margin)	
Umbilical cord measurements (2 dimensions), colour, vessel number, coiling index, abnormalities	
Membrane completeness, insertion, colour, abnormalities	
Sampling: 2 sections of the umbilical cord (fetal and maternal ends); membrane roll including rupture site; periumbilical placental disc, full thickness normal appearing placenta including decidua and samples of placental abnormalities	

Photographs of the placenta are vital. Images should include photographs in the fresh and fixed state, always with a measure. Routine photographs should include the maternal and fetal surfaces of the disc, the umbilical cord and extra placental membranes and the cut surface of the placental disc once serially sliced.

Knowledge of the storage conditions of the placenta is important. The weight of the placenta will reduce over time with storage and increase by about 10% following fixation in formalin [18]. The length of the umbilical cord reduces by 3% 1–2 h and 12% 24–48 h following fixation [19]. The placenta needs to be trimmed of extraplacental membranes and umbilical cord before being weighed as standardized weights only report placenta disc weight [17].

### Microscopic examination of the placenta

Published guidelines for microscopic placental examination are available in standard textbooks [18]. A check list can be found on the PSANZ clinical guidelines [20]. The most important parts of the microscopic assessment are to be systematic and interpretative.

## Major pathological categories associated with an adverse outcome

### Fetal vascular malperfusion

**Fig. 1:**
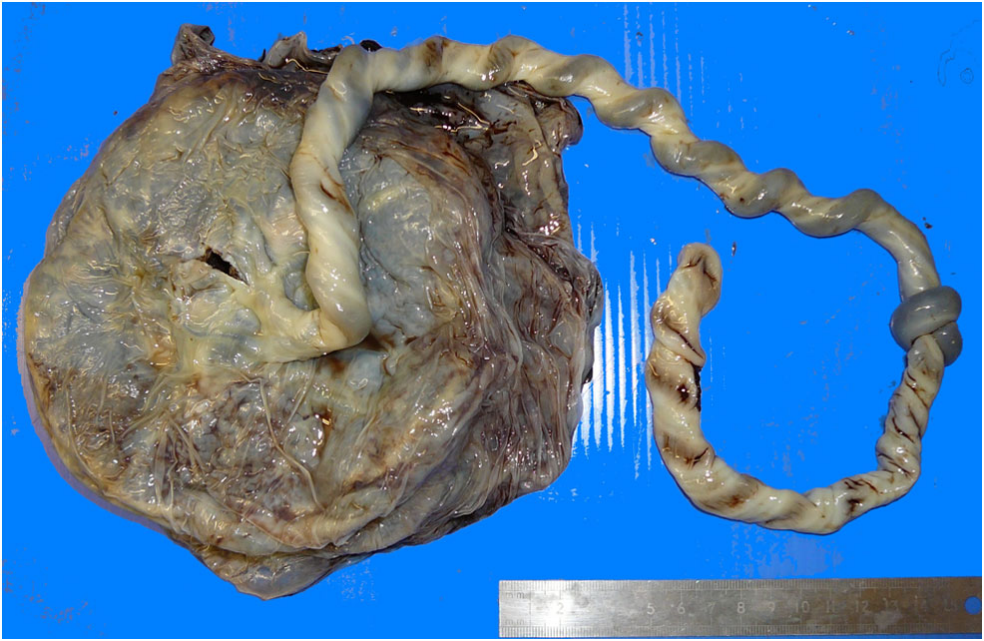
True knot in the umbilical cord resulting in fetal death in utero at 39 weeks. Note the congestion of the vessels on the maternal side of the true knot. The placental disc weighed 460 g. Coiling index 2 coils/5 cm

**Fig. 2:**
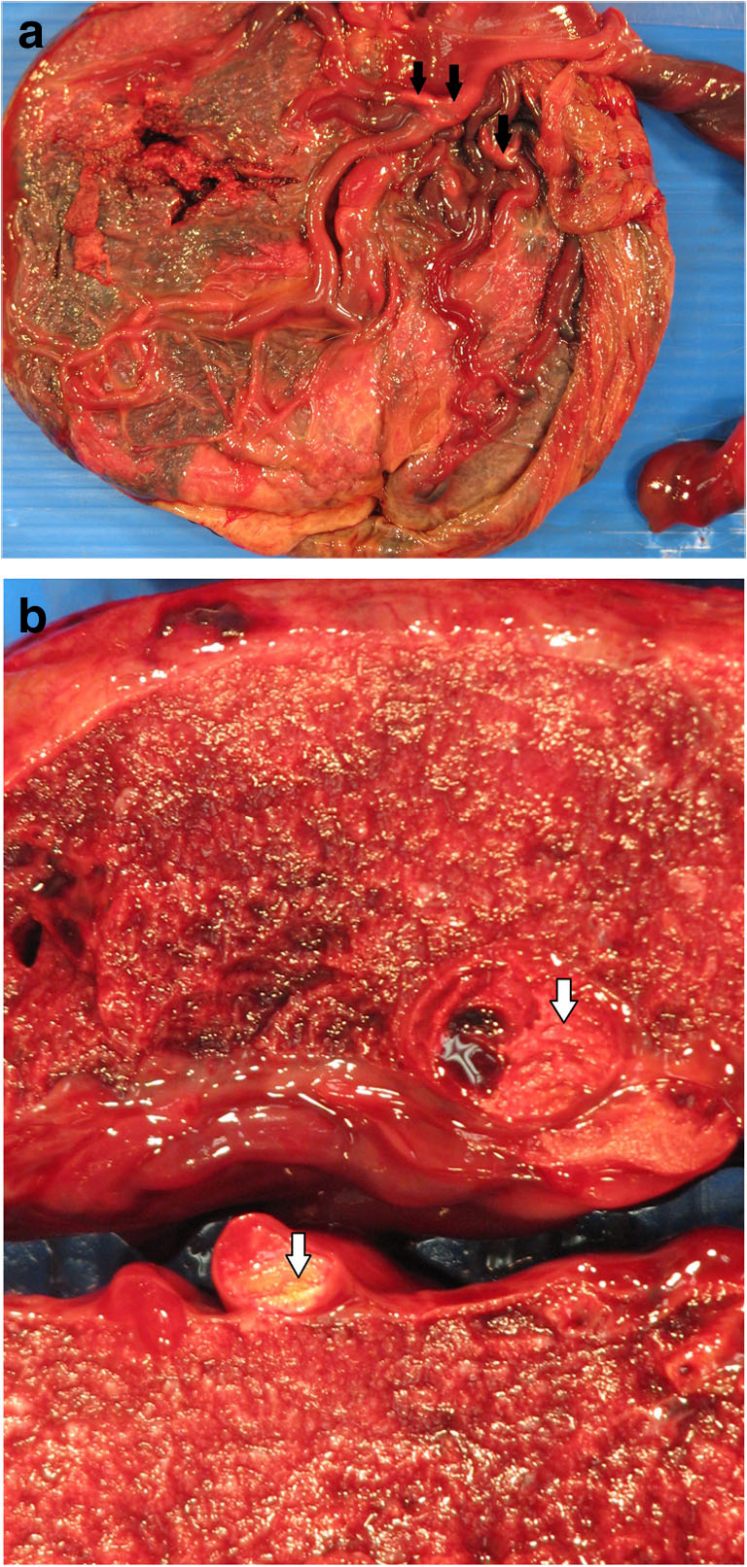
**a** Organizing thrombus in chorionic artery with extension into the stem vessels in association with fetal death in utero at 32 weeks (arrows highlights the thrombus). Note the marginally inserted umbilical cord. **b** Demonstrates a thrombus in chorionic vessel. The placental disc weighed 383 g. Coiling index 4 coils/5 cm

**Fig. 3:**
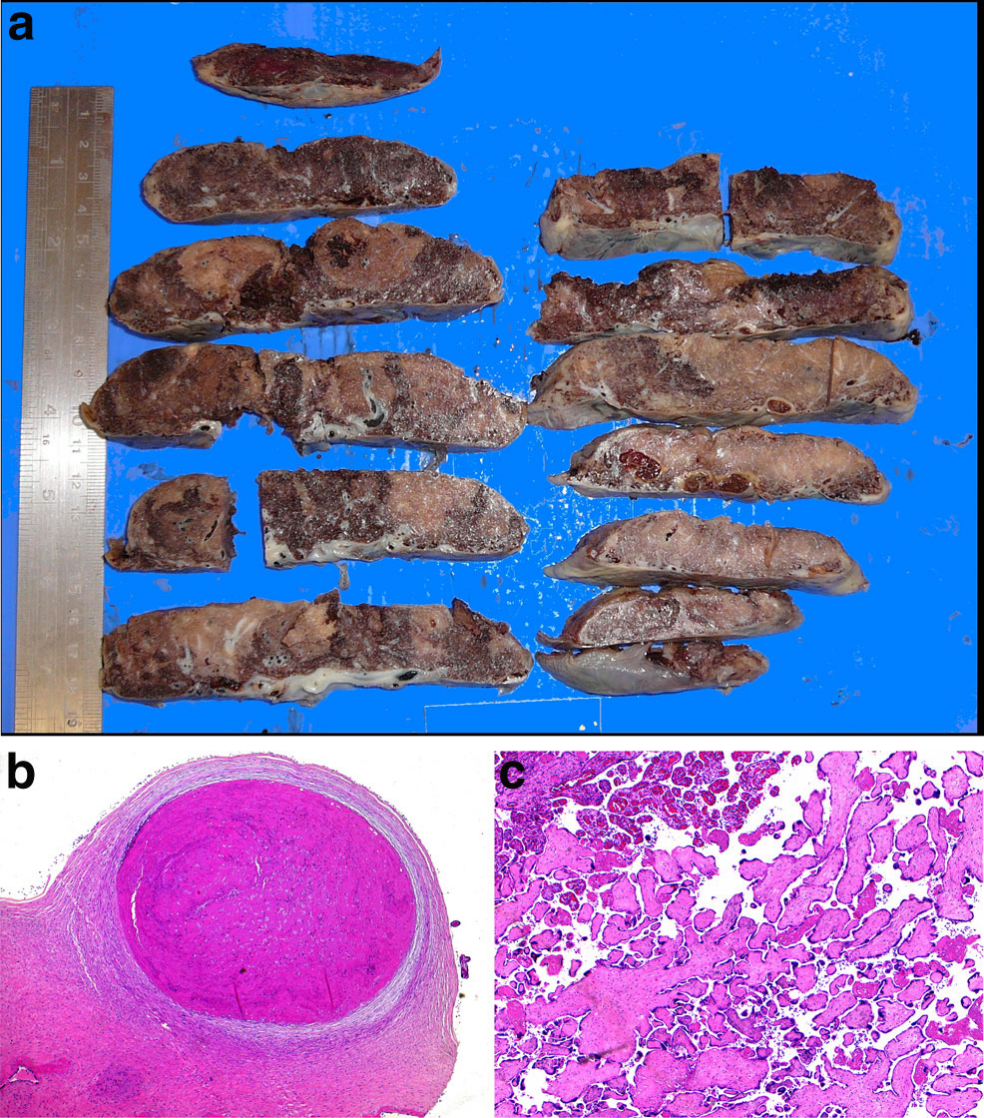
Chorionic vessel thrombosis in 31 + 4 week gestation placenta associated with intrauterine growth restriction. **a** Cut surface of placenta showing pale area involving around 70% of the placenta disc area. **b** Occluding thrombus (H&E, Original magnification ×50). **c** Downstream avascular villi (H&E, Original magnification ×50). The placental disc weighed 263 g. Coiling index 5 coils/5 cm

**Fig. 4:**
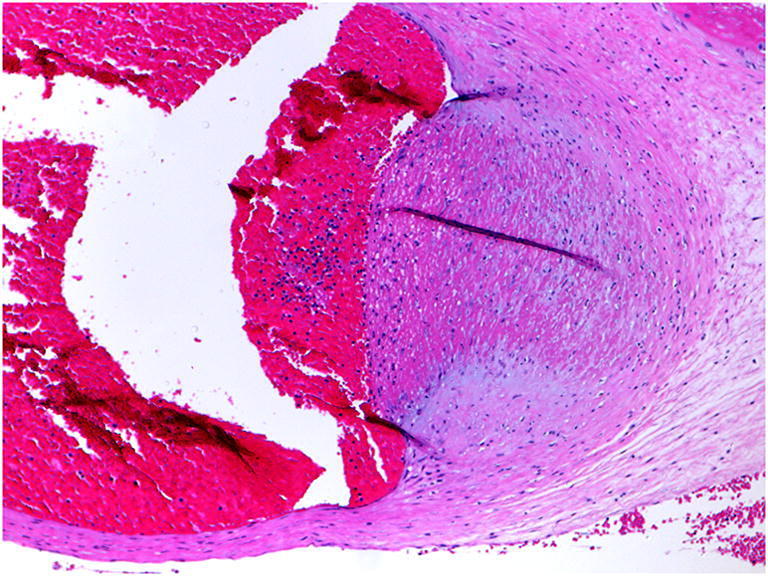
Intramural fibrin deposition (H&E, Original magnification ×100)

**Fig. 5:**
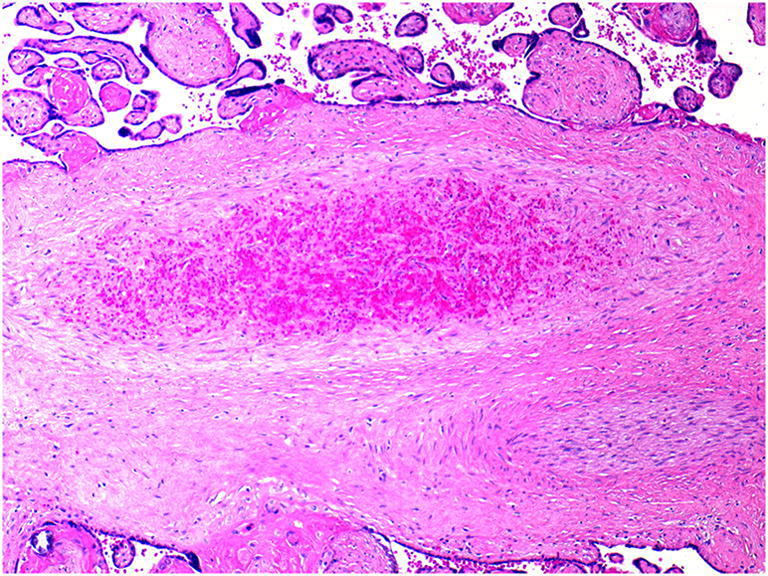
Villous stromal-vascular karyorrhexis due to stem vessel thrombus in 32 week placenta of an infant with intrauterine growth restriction (H&E, Original magnification ×100)

Fetal vascular malperfusion (FVM) (old term fetal thrombotic vasculopathy) is a chronic vaso-occlusive disorder involving the placental villous tree that arises secondary to upstream fetal vascular thrombo-occlusive events [21, 22]. Cessation of blood flow results in involution of affected groups of terminal villi, which eventually become avascular. FMV most commonly reflects vascular stasis from umbilical cord vascular obstruction [23]. Vascular stasis may result from umbilical cord prolapse, hypercoiling (Fig. 1), true cord knots (Fig. 1), excessive cord length, or velamentous insertion, and so these features may be evident macroscopically [23, 24]. There is thus far no conclusive evidence that thrombophilia alone increases the prevalence of FMV [25, 26]. FMV also predisposes affected fetuses/neonates to somatic thromboembolic events [27, 28] and may be a cause of unexpected neonatal encephalopathy and neurologic impairment in term neonates that can predispose to unexpected neonatal death [29]. An autopsy study of third trimester stillbirths also found a correlation of with neuronal injury [30]. A six-fold increase in fetal cardiac abnormalities has been reported in the presence of FVM [31]. Gross manifestations of FMV include thrombi within the umbilical cord vasculature or surface chorionic vessels (Fig. 2a, b) and if long standing, an area (s) of placental disc pallor (Fig. 3a). Such gross findings require histological confirmation. Histological manifestations include intramural fibrin deposition (old term intimal fibrin cushion, Fig. 4) or chorionic or stem vessel obliteration (old term fibromuscular sclerosis, Fig. 3b) affecting large fetal vessels, villous stromal-vascular karyorrhexis (old term hemorrhagic endovasculitis) (Fig. 5) and avascular chorionic villi (Fig. 3c) [22, 32]. High grade FMV is regarded as being more clinically significant and can be associated with sudden deterioration in the first 24 h and perinatal death [17, 22, 32–34]. The early changes of the FMV are those of villous stromal-vascular karyorrhexis and occur within six hours, followed by involution of the stem vessel which is seen by 48 h, and finally avascular villi which takes around two weeks. These changes are identical to those seen in association with intrauterine demise but usually only affect a segment of the placenta [35]. FVM can occur at any gestation but is most commonly seen in association with third trimester stillbirths.

### Maternal vascular malperfusion including placental infarction

**Fig. 6:**
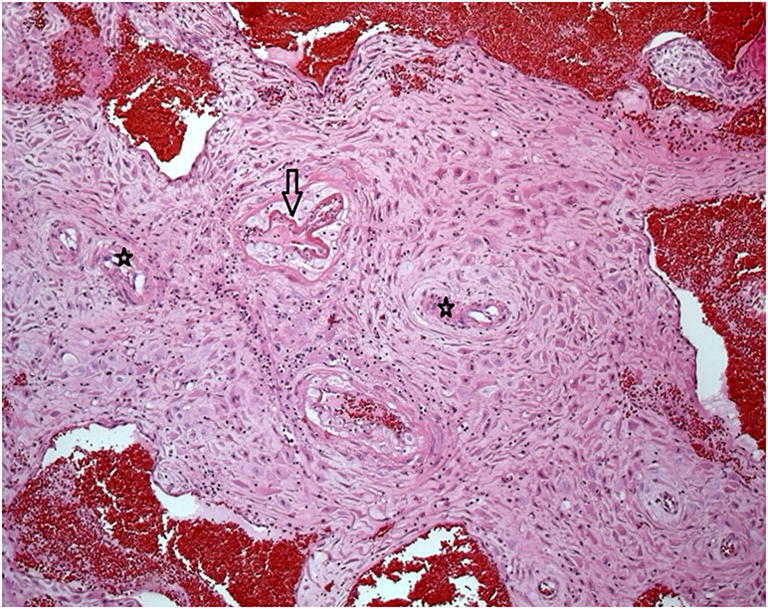
Decidual arteriopathy: Decidual vessels shows influx of foamy macrophages and focal fibrinoid necrosis (arrow). Note also persistence of smooth muscle in some vessels (stars)

**Fig. 7:**
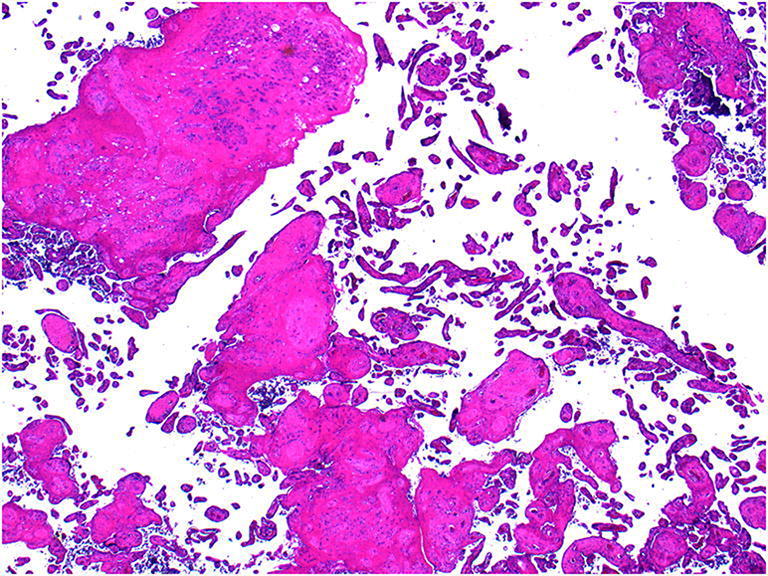
Accelerated villous maturation: 34 week gestation placenta showing smaller terminal villi and increased perivillous fibrin deposition for the stated gestation. The placental disc weighed 270 g (H&E, Original magnification ×25)

**Fig. 8:**
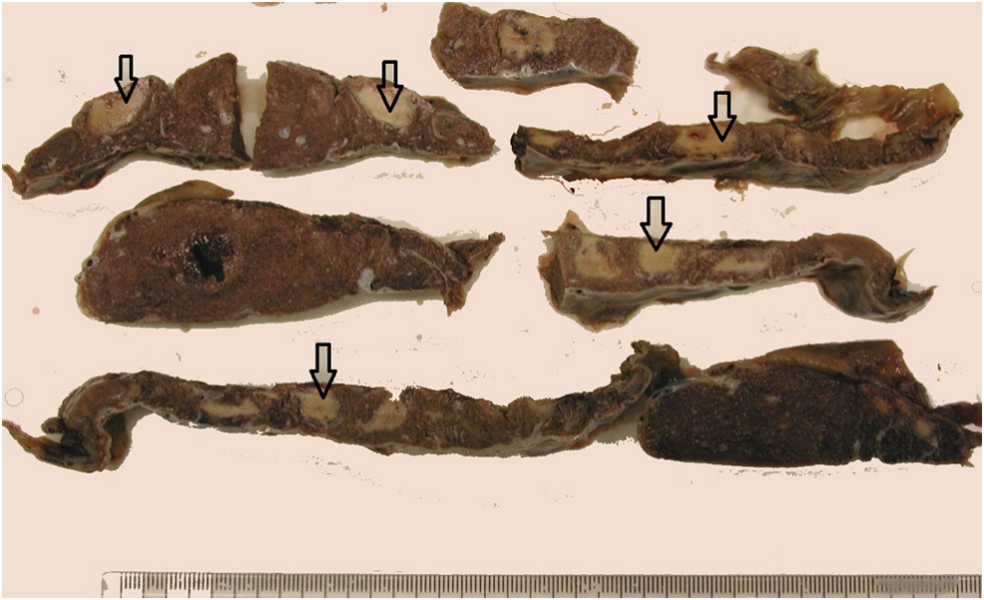
Slices of placenta showing multiple placental infarcts of varying ages from a fetal death in utero at 35 weeks (arrows high light older infarcts). The mother was found to have a thrombophilia. The placental disc weighed 323 g. Coiling index 1 coil/5 cm

**Fig. 9:**
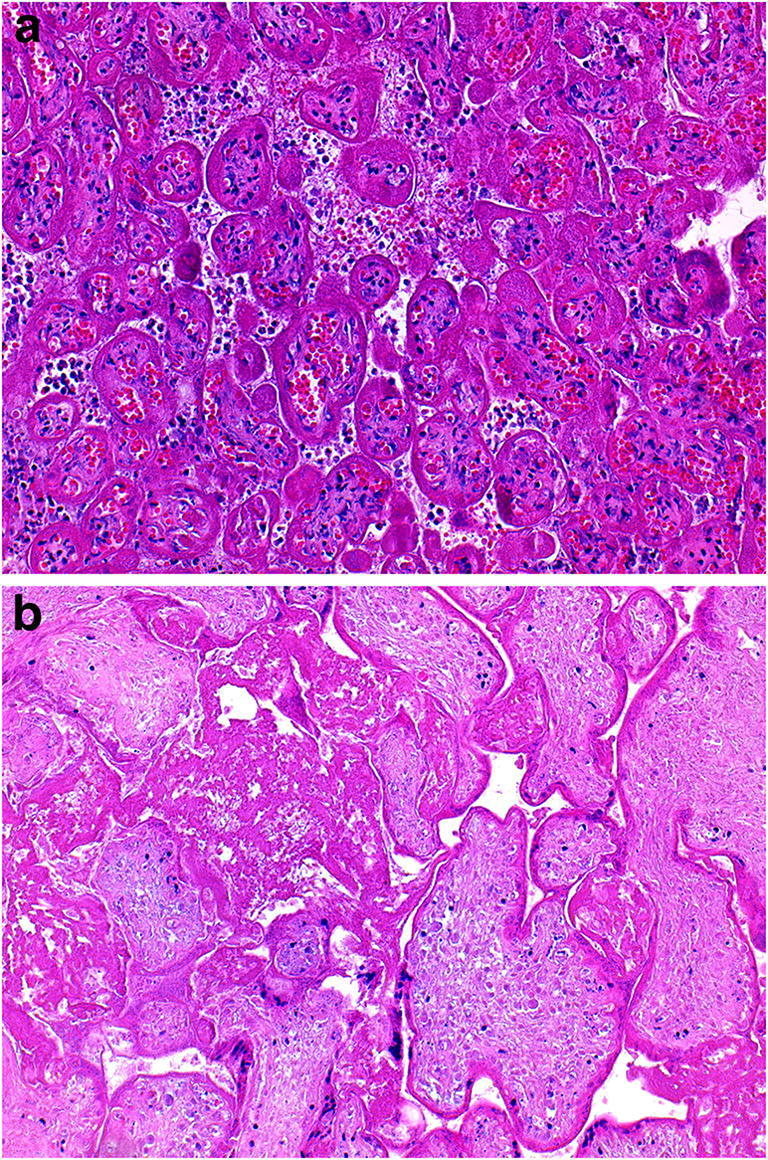
**a**.Earlier placental infarct from a placenta delivered at 31 weeks with a history of pre-eclampsia for 3 days and intrauterine growth restriction. 25% of the disc was infarcted. Photomicrograph shows collapse of villi and presence of neutrophils in the intervillous space. The placental disc weighed 407 g. Coiling index 3 coils/5 cm (H&E, Original magnification ×200); **b** Old placental infarct from a placenta delivered at 30 weeks with a history of hypertension and pre-eclampsia and intrauterine growth restriction. 30% of the disc was infarcted. Photomicrograph shows perivillous fibrin deposition and absence of basophilia of the trophoblasts lining the villi. The placental disc weighed 153 g. Coiling index 1 coil/5 cm. (H&E, Original magnification ×200)

During the course of pregnancy maternal arteries undergo physiological conversion, a process by which trophoblastic invasion of the uteroplacental vessels converts relatively small caliber muscular arteries to large caliber and low resistance vascular channels [34]. Abnormalities of this process results in decidual arteriopathy and a spectrum of pathological features affecting the maternal vasculature, including acute atherosis (Fig. 6), mural hypertrophy of the membrane arterioles and muscularisation of basal plate arteries (Fig. 6) [36]. Villous malperfusion results in accelerated villous maturation (Fig. 7). This is a process in which histological features of distal villous hypoplasia and Tenney-Parker changes (syncytial knot formation) impart a histological appearance of development and maturation which is too advanced for the actual gestational age. In addition, such placentas are often small for gestational age and have an increased fetoplacental weight ratio [37]. Complete cessation of maternal vascular perfusion to a region of the placenta results in infarction. Large, or multiple small, infarcts involving greater than 30% of the non-peripheral portion of the placenta, or infarcts in premature placentas, are markers for significant maternal vascular disease, especially hypertension. These placentas are often hypoplastic, weighing less than 10th centile for the stated gestational age. Features of decidual arteriopathy, accelerated villous maturation (Fig. 7) and placental infarction (Fig. 8) are seen in pregnancies affected by preeclampsia and gestational hypertension, lupus erythematosus, and in women with lupus anticoagulant and antiphospholipid antibodies. Dating of placental infarcts is not accurate. Macroscopically they change in color from red to cream over time. Microscopically, the collapse of villi and presence of neutrophils implies an infarct of at least 12 h duration (Fig. 9a) while an infarct of greater than 36 h with show neutrophil karyorrhexis. Perivillous fibrin deposition and the total absence of nuclear basophilia of the trophoblasts is seen in old infarcts of weeks duration (Fig. 9b). Maternal vascular malperfusion is an important cause of fetal growth restriction and preterm delivery that can result in poor Apgar scores, cerebral palsy and perinatal death [33, 38]. Changes of maternal vascular malperfusion commence early in gestation but are most commonly manifest as a cause of stillbirth in the late second and third trimesters. It can also provide evidence of underlying maternal vascular disease in unexpected maternal convulsions or stroke.

### Placental hematomas and thrombi

**Fig. 10:**
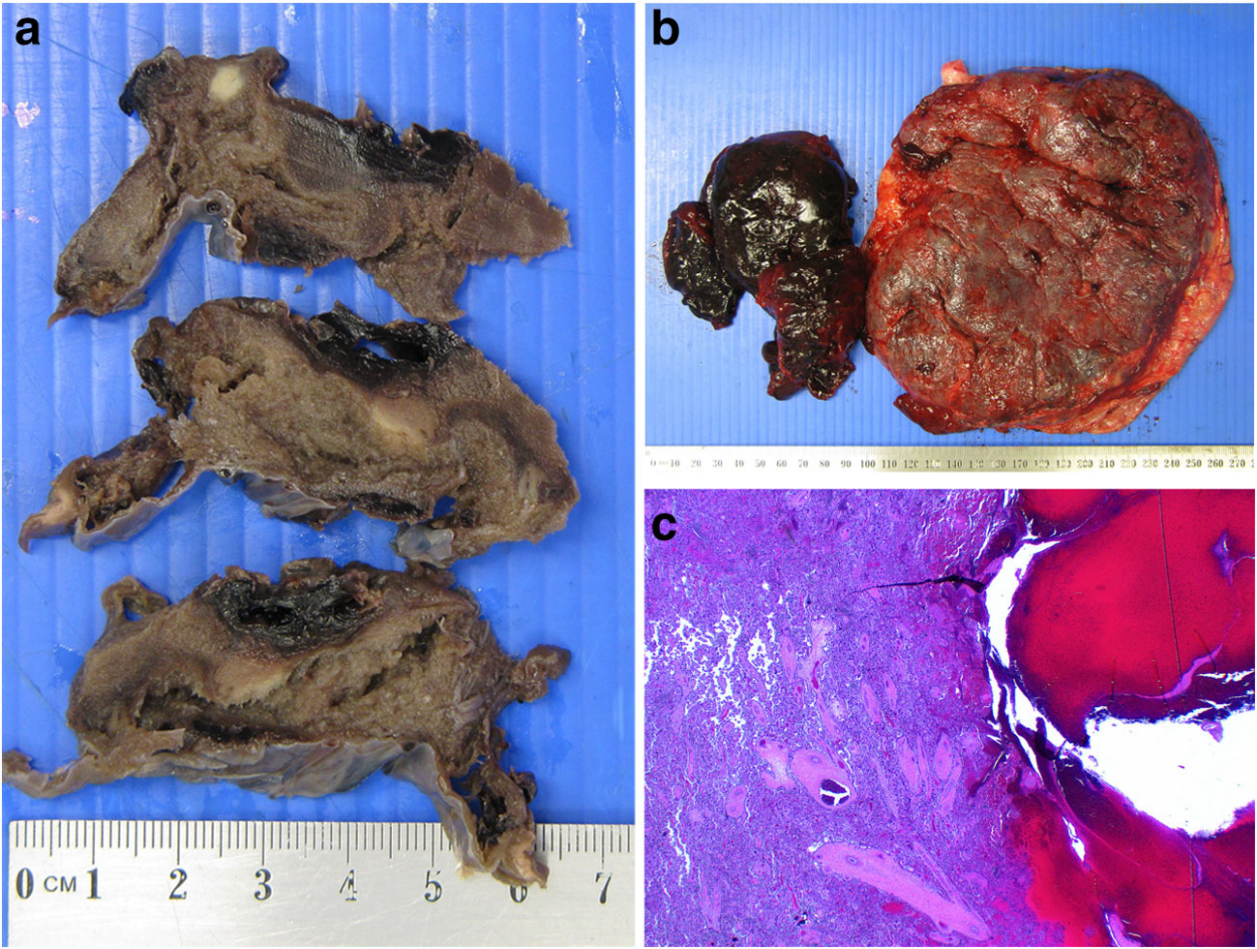
**a** Chronic retroplacental hemorrhage resulting in fetal death at 26 weeks. The placental disc weighed 85 g. Coiling index 1 coil/5 cm. **b** Acute retroplacental hemorrhage: Stillbirth at 37 + 5 weeks. The placental disc weighed 425 g with a loosely adherent clot measuring 100x100x10mm. Note no obvious compression of the disc. **c** Retroplacental hemorrhage in a 30 week gestation placenta associated with intrauterine growth restriction (900 g). The mother had severe pre-eclampsia. The placental disc weighed 280 g. Note the disc compression, intervillous hemorrhage and early villous infarction (H&E, Original magnification ×12.5)

Hematoma or thrombus formation may occur at various anatomical localities within the placenta, and include retroplacental hematomas, marginal hematomas, intervillous thrombi, subchorionic thrombi, and subamniotic hematomas [9]. Marginal hematomas are crescent -shaped clots located at the margin of the placental disc. Generally, the most clinically significant are retroplacental hematomas as they are the morphological substrate for the clinical syndrome of placental abruption. The usual clinical criteria for a diagnosis of abruption includes (i) evidence of retroplacental clot(s); (ii) abruption diagnosed on prenatal ultrasound; or (iii) vaginal bleeding accompanied by nonreassuring fetal status or uterine hypertonicity. These criteria are however weak [39]. In the classical case, a large laminated blood clot adherent to the maternal surface of the placenta, is seen compressing the overlying placental parenchyma (Fig. 10a). Such findings require hours or days to develop. A more acutely developing retroplacental hematoma may not be accompanied by such changes and may be non-adherent to the placenta (Fig.10b). In such situations, the pathologist is reliant on information provided by the clinician. Morphological clues to the occurrence of a significant acute retroplacental hematoma include a fixed depression in the maternal surface, histological findings of decidua basalis hemorrhage with necrosis and inflammation, adjacent intravillous hemorrhage, and in some situations, early villous infarction (Fig. 10c). After two to three days pigmented histiocytes may be seen histologically in the membranes or decidua [40]. Of clinically diagnosed cases, the sensitivity and specificity for a histologic confirmation of abruption has been reported as 30.2% and 100%, respectively [39]. The causes of retroplacental hemorrhage are varied, but in a significant proportion of cases, are related to abnormalities of the vascular bed arteries as described under maternal vascular malperfusion, and should be specifically looked for. Other non-traumatic causes include amniotic fluid infection. Retroplacental hematoma may occur as a result of maternal assault or traffic accident, with subsequent abruption that may occur hours or days later and has been previous reviewed by others [41]. In general, differentiating a traumatic versus a non-traumatic cause for placental abruption in the absence of clinical history is challenging given abruption can occur independent of trauma. If someone is found to have abnormalities of the uterus known to be associated with abruption or a history of past abruption, is inflicted trauma more likely to precipitate abruption? Given the sensitivity of histologically confirming abruption it is not surprising that exact timing of a placental abruption is also often not possible. Placental “silent” abruption may also occur with or without a mother being aware of any clinical symptoms.

**Fig. 11:**
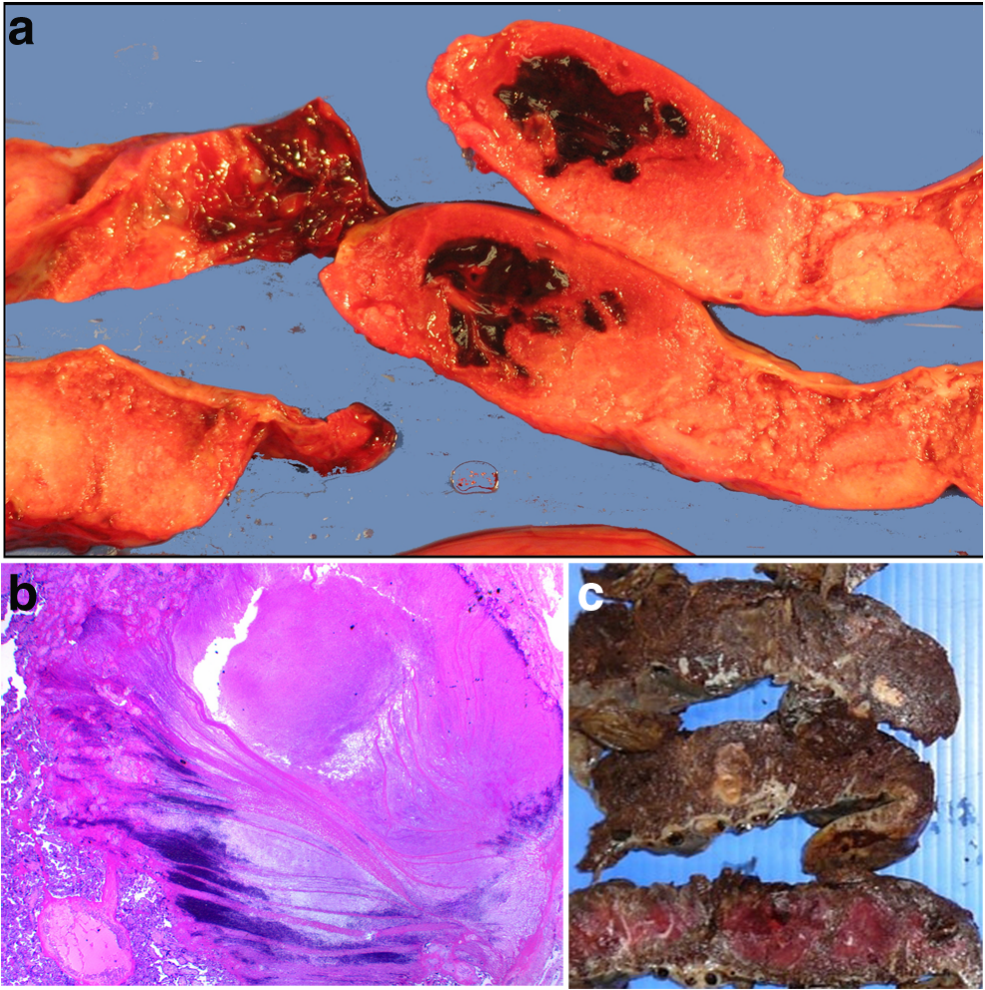
Intervillous thrombi in case of fetomaternal hemorrhage at 40 weeks gestation. Fetomaternal hemorrhage of 150.5mls. The placental disc weighed 485 g. **a** Macroscopic intervillous thrombi **b** microscopic appearance of intervillous thrombus (H&E, Original magnification ×12.5) **c** Macroscopic old intervillous thrombus from a term placenta

Intervillous thrombi are blood clots occurring within the placental parenchyma which result in displacement and compression, and sometimes infarction, of the surrounding chorionic villi. They are formed from a mixture of fetal and maternal blood. When fresh they appear red and soft and when old firm and cream macroscopically (Fig. 11 a, b and c). The significance of intervillous thrombi is as an indicator of fetal bleeding into the intervillous space and hence into the maternal circulation [9]. Massive intervillous thrombi are frequently associated with poor perinatal outcome. In the event of significant neonatal anemia, stillbirth or other adverse neonatal outcome, the Kleihaur-Betke acid-elution test which measures the amount of fetal hemoglobin in the maternal circulation may be helpful in assessing the presence, chronicity and severity of fetal to maternal hemorrhage.

**Fig. 12:**
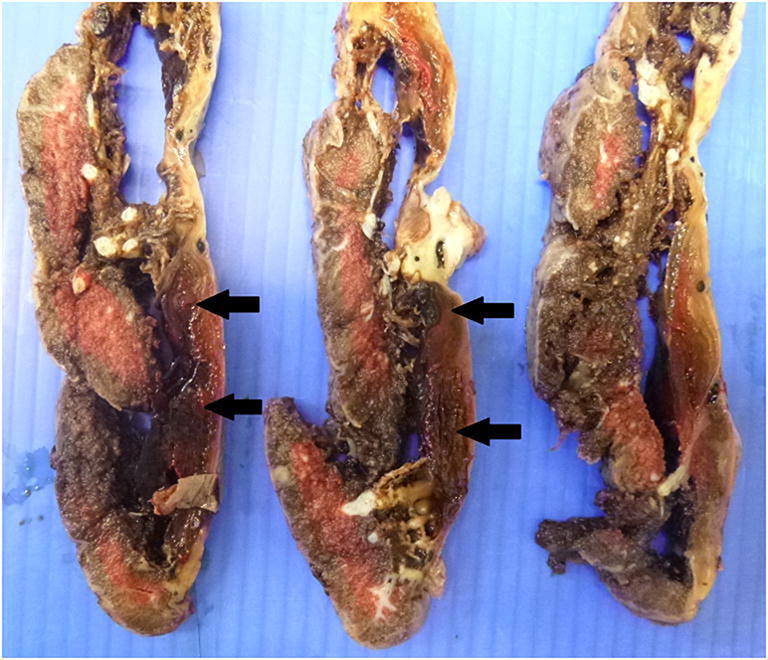
Subchorionic hematoma (black arrows) from a placenta delivered at 40 + 2 weeks with a history of decreased fetal movements and poor CTG: 40 + 2. The placental disc weighed 391 g, subchorionic thrombus occupied 30% of disc area

Subchorionic hematomas form in small quantities physiologically as a result of backwards deflection and eddying of maternal blood in this location [9]. Massive subchorionic hematomas involving more than 50% of the placental disc are associated with poor reproductive outcome, including preterm delivery, abortions, intrauterine fetal growth restriction and intrauterine fetal death (Fig. 12). The Breus’ mole is an overlapping entity consisting of a massive subchorionic hematoma which is diffusely nodular and which forms blood-filled protrusions when viewed from the fetal aspect of the placenta [9]. It is associated with circumvallation, neonatal demise, monosomy X, and maternal diseases such as diabetes mellitus and hypertension.

Subamniotic hemorrhages or hematomas are free lying collections of blood located between the amnion and chorion on the fetal plate of the placenta. They arise due to trauma to the surface vessels as a result of traction on the umbilical cord during delivery and are common in specimens from cesarean deliveries. They are usually of no clinical significance.

### Meconium staining

Meconium is normally sterile but contains high levels of bile acids and lipases. Meconium passage in utero has traditionally been considered an indicator of acute fetal distress, especially in relation to an asphyxial event [42]. However, in the late third trimester, meconium staining is present in approximately 20% of placentas [43] and, conversely, many instances of fetal hypoxia and distress are unaccompanied by meconium passage [44].

**Fig. 13:**
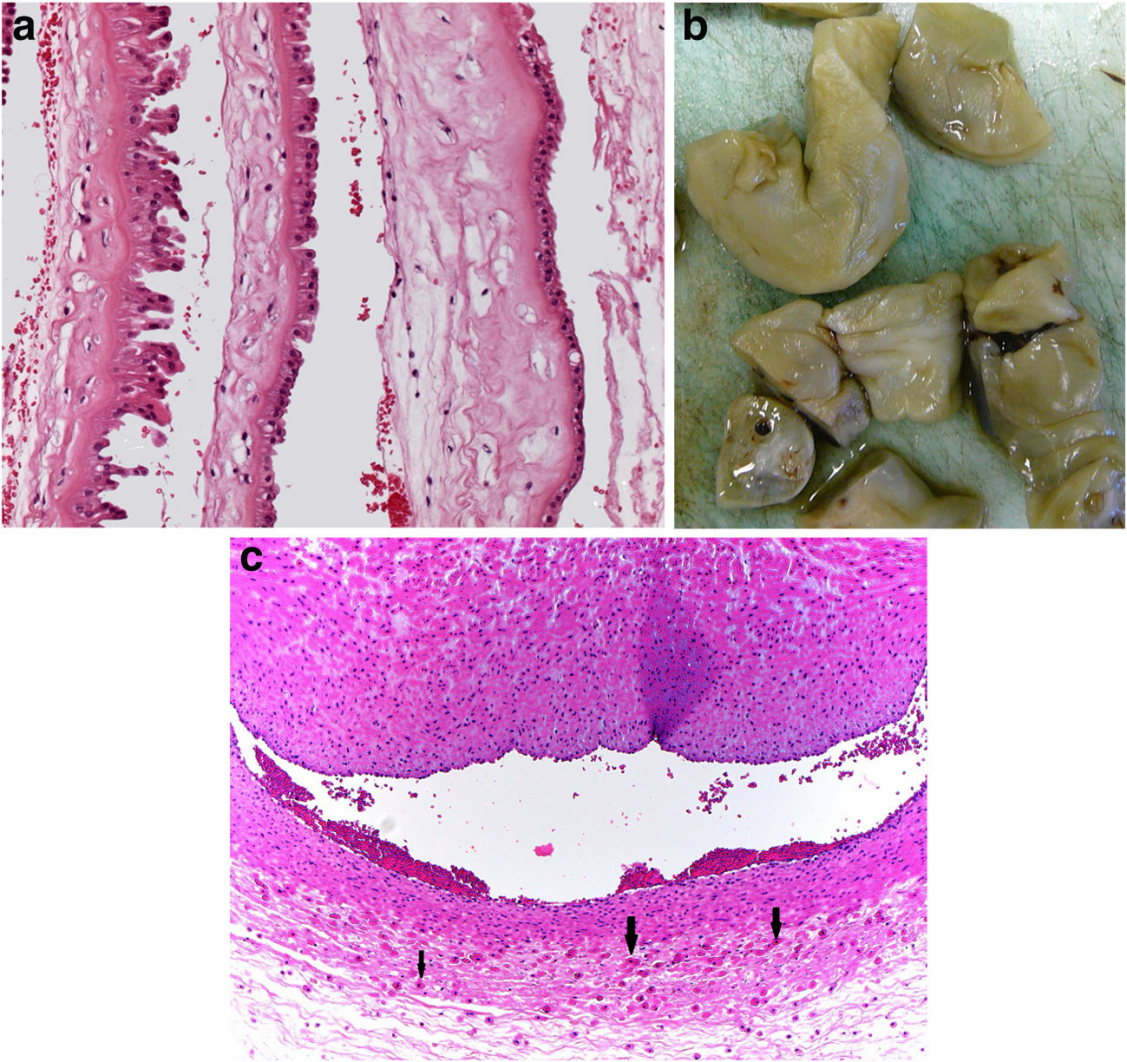
**a** Meconium induced damage of the amniotic epithelium in a 36 week gestation placenta of an infant who died subsequent to meconium aspiration syndrome (H&E, Original magnification ×200). **b** Meconium induced myonecrosis in the umbilical cord from a placenta delivered at 41 + 3 weeks. The infant became unexpectedly hypoxic, developed seizures and multi-organ failure. The placental disc weighed 413 g. Coiling index 1 coil/5 cm. Meconium stained umbilical cord. **c** myonecrosis of the umbilical artery. Note the necrotic myocytes (black arrows) are concentrated towards the edge of the cord (H&E, Original magnification ×200)

The clinical significance of meconium relates to the time interval from meconium passage to delivery. Meconium pigment may be identified in amniotic macrophages as early as one hour after discharge, and after 4–6 h may also be identified in chorionic macrophages [45]. Degenerative change, necrosis or loss of the amniotic epithelium is common (Fig. 13a). Acute meconium staining at or near the time of delivery may have no clinical relevance. Conversely, prolonged meconium staining of the placenta indicates a high risk to the fetus of meconium aspiration syndrome [46], perinatal asphyxia, cerebral palsy, or other central nervous system deficits [47]. The umbilical vein and chorionic surface vessels undergo contraction and segmental mural necrosis with degeneration of the medial myocytes (meconium induced vascular necrosis (myonecrosis)) (Fig. 13b and c). This can be a cause of unexpected poor Apgar scores, convulsions and perinatal death [48–50].

### Abnormalities of umbilical cord insertion

**Fig. 14:**
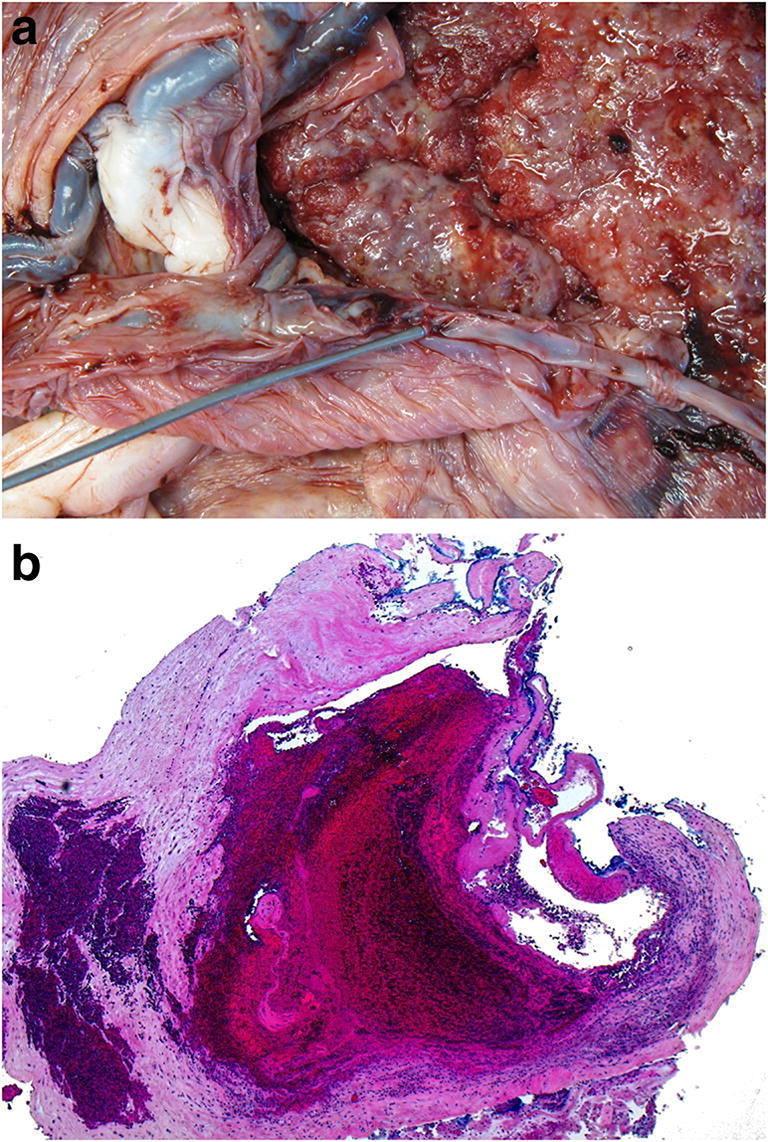
Tear in membranous vessel from a placenta delivered vaginally at 40 + 2 weeks. Severe birth asphyxia and neonatal anemia. The placental disc weighed 600 g. Coiling index 1 coil/4 cm **a** Tear in a membranous vessel at rupture site (marked by metal probe). **b** Histology of membranous vessel at site of rupture showing acute inflammation and necrosis of the vessel wall (H&E, Original magnification ×50)

The insertion of the umbilical cord may be normally central, paracentral or eccentric. Cords inserting less than one centimeter from the nearest edge of the placental disc (marginal insertion) and into the membranes (velamentous insertion) are more common in multiple pregnancies, in association with single umbilical artery and small placental discs. Velamentous cord insertions result in the presence of membranous blood vessels which run along the free placental membranes unprotected by Wharton’s jelly. Such vessels are vulnerable to injury and are at risk of thrombosis or disruption during labor leading to massive fetal blood loss (Fig. 14a and b). Velamentous vessels located over the cervical os constitute the serious condition of vasa previa, and pose the risk of rupture, hemorrhage and exsanguination during vaginal delivery [9].

### Amniotic fluid infection (maternal and fetal inflammatory responses)

**Fig. 15:**
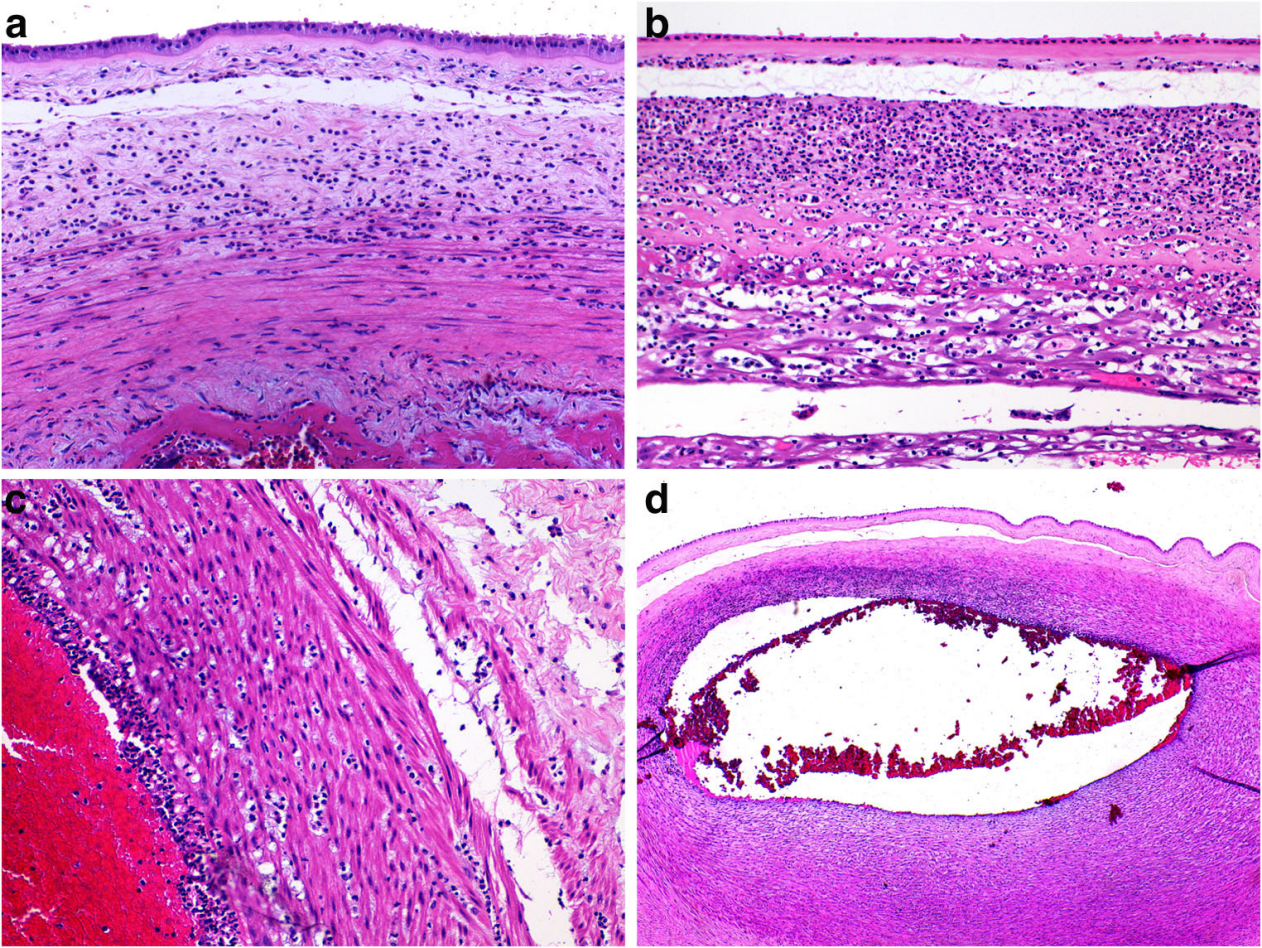
Ascending infection. High vaginal swab grew E.coli from a placental delivered vaginally at 38 + 2 weeks**. a** Neutrophils in the subchorionic fibrin extending into the placental plate (H&E, Original magnification ×200). **b** Acute chorioamnionitis (maternal inflammatory response grade 2, stage 3) (H&E, Original magnification ×200). **c** acute vasculitis (arteritis) with funisitis (fetal inflammatory response grade 1, stage 2) (H&E, Original magnification ×200). **d** Acute vasculitis of a chorionic plate vessel. Note the concentration of neutrophils on the amniotic cavity side of the vessel (H&E, Original magnification ×50)

**Fig. 16:**
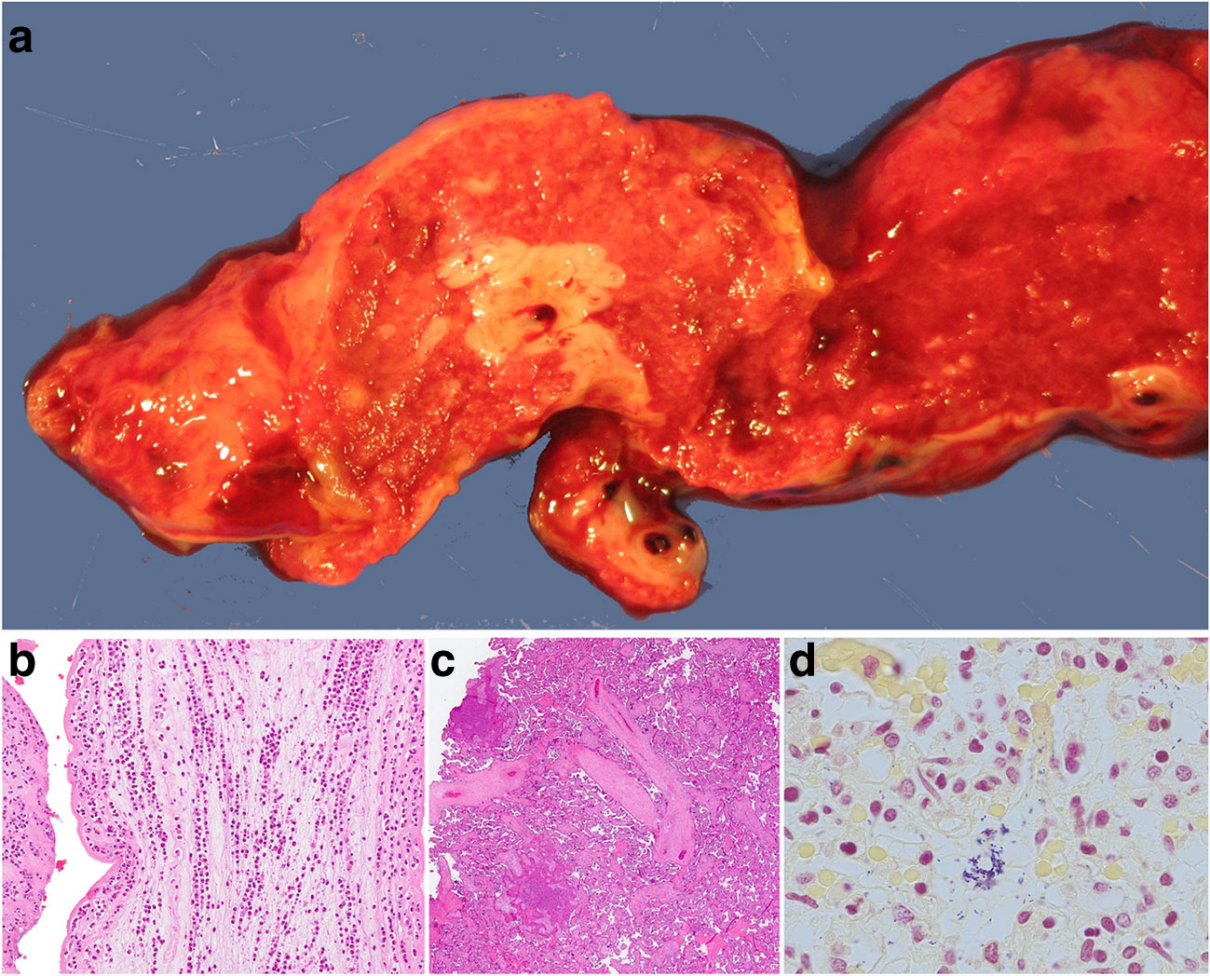
*Listeria monocytogenes*. Mother presented at 30 weeks gestation with a fever of 38 °C. **a** Multiple abscesses evident within the placental parenchyma. **b** Acute chorioamnionitis (maternal inflammatory response grade 2, stage 3) (H&E, Original magnification ×200). **c** Two microabscesses (black arrows) at low power (H&E, Original magnification ×50) and high power (H&E, Original magnification ×400). **d** Gram stain demonstrated gram positive bacilli in the intervillous space and membranes (H&E, Original magnification ×1000)

Acute chorioamnionitis (maternal inflammatory response) is defined histologically by the presence of acute inflammatory cells within the fetal membranes. It indicates sepsis within the amniotic cavity usually due to an ascending bacterial infection. Macroscopically this may result in the membranes appearing opaque and/or slimy and the placenta being malodorous. Frequently however no specific features are seen macroscopically. There is also poor correlation of histological acute chorioamnionitis with “clinical” acute chorioamnionitis, as defined by maternal fever, leukocytosis, uterine tenderness, or maternal or fetal tachycardia. In addition, women with histological acute chorioamnionitis may not have clinical manifestations of clinical chorioamnionitis [51]. Histological chorioamnionitis has still, however, been shown to be both sensitive and specific for infection and is the gold standard against which other clinical predictors of infection are measured. While correlation with microbiological culture is important, in the age of widespread antibiotic usage, it is not uncommon for the culture result to be negative [52]. Routine histochemical stains for bacterial (Gram stain) and fungal organisms (Silver stain such as Grocott stain or PAS stain) should be performed on the section of inflamed extraplacental membranes appreciating that they have variable sensitivity and specificity. Identification of the pathogenic species may also be achieved using a range of molecular genetic techniques, including multiplex real-time PCR assay, multiplex real-time PCR assay, hybridization probes, DNA microarrays 22 and 16S rRNA gene-based PCR and sequence analysis, including on archival material [53, 54]. Each has their advantages and disadvantages over culture. The mother and fetus both contribute to the inflammatory response in amniotic infection. The maternal response begins with the emigration of neutrophils from decidual vessels and intervillous spaces [9] (Fig. 15a). The neutrophils then spread through the chorion and amnion in response to chemotactic factors in the amniotic fluid (Fig. 15b). The fetal response (fetal inflammatory response) occurs later than the maternal response, but only if the fetus remains alive and is older than 20 weeks in gestational age. It consists of vasculitis within the umbilical cord affecting the umbilical vein, then the arteries (Fig. 15c), and the chorionic plate surface vessels (Fig. 15d). Table 3 provides a summary of staging and grading of maternal and fetal inflammatory response and their approximate timing. Although grading and staging do have some significance in relation to neonatal outcome, the most important features of ascending infection are the identity of the infectious agent and whether there is a fetal response, e.g. Trichomonas spp. often elicits a heavy and dense neutrophilic infiltrate yet has little effect on neonatal wellbeing. Conversely, Group B streptococcal infection is one of the most virulent perinatal infections, yet intra-amniotic infection and neonatal sepsis may occur without identifiable histological chorioamnionitis [55]. Nonetheless, specific histological findings have been shown to have prognostic significance in relation to fetal outcome. Necrotizing chorioamnionitis (grade 2, stage 3 maternal inflammatory response) is a late complication of amniotic inflammation and is associated with an increased risk of perinatal death, intraventricular hemorrhage, retinopathy of prematurity and preterm delivery [56] and should be specified in the pathology report [57]. Necrotizing funisitis (grade 2, stage 3 fetal inflammatory response) is a severe pattern of umbilical cord inflammation characterized by yellow-white calcific rings around the cord vessels. It is rare but has a high association with significant fetal and neonatal morbidity and mortality [33]. When acute chorioamnionitis is associated with intervillous inflammation/ abscess formation it is commonly due to *Listeria monocytogenes* (Fig. 16a–d). In addition to Listeria, Campylobacter, Chlamydia, Francisella, Coccidioides and Arthrobacter spp. are also implicated [58].

**Table 3:** A summary of staging and grading of maternal and fetal inflammatory responses and their approximate timing in ascending intrauterine infection [17, 59]

Maternal inflammatory response	
Stage 1 (6–12 h) – acute subchorionitis or chorionitis Stage 2 (12–36 h) – acute chorioamnionitis: polymorphonuclear leukocytes extend into fibrous chorion and/or amnion Stage 3 (>36 h) – necrotizing chorioamnionitis: karyorrhexis of polymorphonuclear leukocytes, amniocyte necrosis, and/or amnion basement membrane hypereosinophilia	Grade 1 – not severe as defined Grade 2 – severe: confluent polymorphonuclear leukocytes or with subchorionic microabscesses.
Fetal inflammatory response	
Stage 1 – chorionic vasculitis or umbilical phlebitis Stage 2 – involvement of the umbilical vein and one or more umbilical arteries Stage 3 – necrotizing funisitis	Grade 1 – not severe as defined Grade 2 – severe: near-confluent intramural polymorphonuclear leukocytes with attenuation of vascular smooth muscle

From a practical point of view, there are important questions that require answers when a potentially inflamed placenta is submitted for pathological examination [59]. Was the fever, abdominal tenderness or maternal leukocytosis due to chorioamnionitis or some other condition, e.g. cervical incompetence, maternal vascular disease, administration of certain drugs, or chronic placental abruption? Were the symptoms and signs in the newborn, such as growth retardation, abnormal neurological status, cytopenia and organomegaly, explained by infection, or should some other cause, such as metabolic or congenital heart disease, be considered? As certain infectious organisms, such as *Candida albicans*, *Listeria monocytogenes* and Treponema pallidum, are treatable; histology or microbiology may be able to confirm the cause of infection due to one of these organisms. Amniotic fluid infection resulting in fetal death is more likely to occur at 21–24 weeks, resulting in preterm delivery than at term [60].

Infection can also spread to the placenta hematogenously. If the organism is bacterial the mother is usually clinically unwell. Maternal blood cultures should be performed. Common organisms that can spread to the placenta hematogenously include Listeria, *Staphylococcus aureus* and the TORCH (toxoplasmosis, rubella, cytomegalovirus, herpes simplex) viruses, including Zika virus [61, 62]. Rarely amniotic fluid infection can occur as result of an invasive procedure such as amniocentesis or potentially retrograde seeding from peritoneal cavity from fallopian tubes [63, 64].

### Chronic villitis

**Fig. 17:**
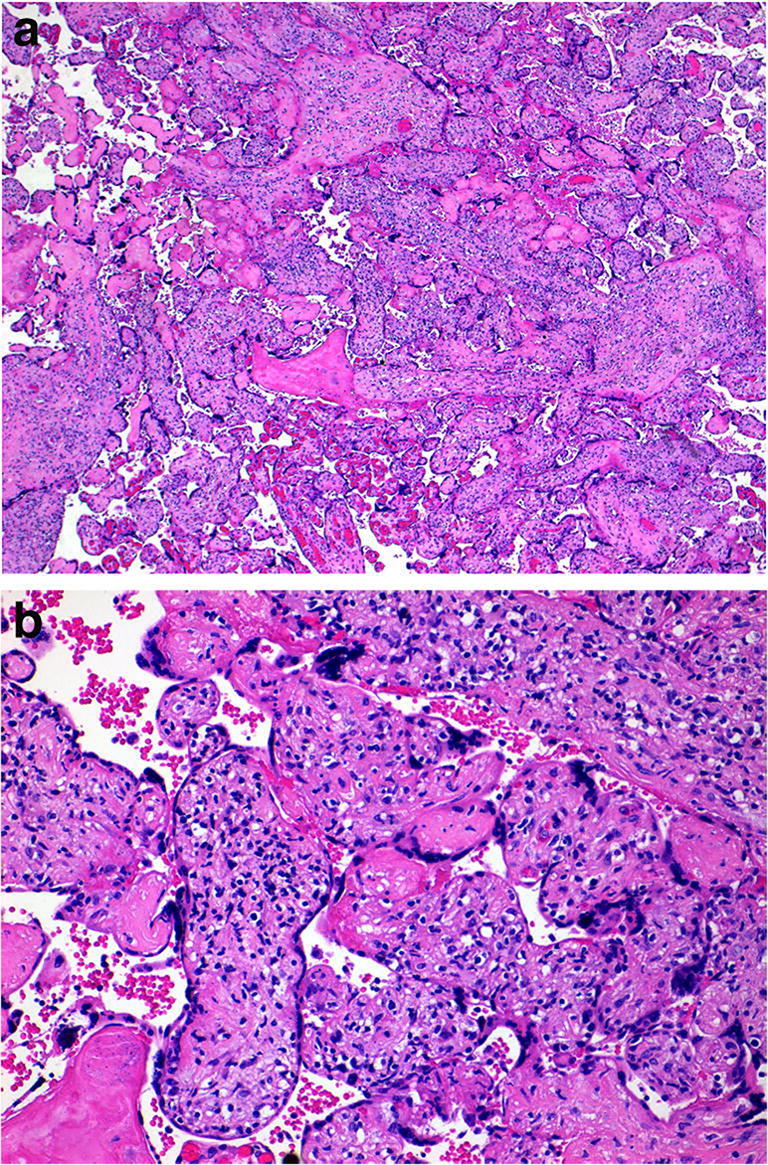
High grade chronic villitis of unknown etiology (VUE) in a placenta delivered at 37 + 2 weeks. The infant showed asymmetrical IUGR (2.63 kg) with abdominal circumference on 2nd percentile. The placental disc weighed 349 g. Coiling index 1 coil/5.5 cm. **a** infiltrate of villi by inflammatory cells. Note the avascular villi on the right edge of the photomicrograph indicative of old fetal vessel occlusion (H&E, Original magnification ×50) **b** infiltrate of villi by lymphocytes and histiocytes and loss of vascularity of the villi (H&E, Original magnification ×200)

Chronic villitis is now regarded as being either non-infectious or infectious in origin. Villitis of unknown etiology (VUE) is a histopathologic diagnosis characterized by an infiltrate of maternal lymphocytes and histiocytes (without plasma cells) affecting the chorionic villi (Fig. 17a and b) [9]. Current evidence favors a theory that VUE is due to disrupted pregnancy immune regulation caused by a failure of maternal-fetal/placental tolerance [65], essentially representing a maternal type 1 delayed-type hypersensitivity allograft reaction. The majority of lymphocytes are maternally-derived CD8-positive cytotoxic T lymphocytes [66]. The non -lymphoid component of the infiltrate consists of resident fetal villous macrophages (Hofbauer cells) which function as antigen-presenting cells, and perivillous monocyte- macrophages of maternal origin. Fetal Hofbauer cells have been shown to proliferate in VUE and become activated as evidenced by the up regulation of class II major histocompatibility complex antigen expression [67]. Epithelioid histiocytes and multinucleate giant cells may be identified histologically and it can be associated with or without obliterative vasculopathy of stem villous vessels (Fig. 17a) [9]. VUE is associated with a placental hypoplasia but no obvious macroscopic lesion. Clinically it is associated with fetal growth restriction, low Apgar scores due to neonatal encephalopathy and stillbirth [33, 65]. Stillbirth usually occurs in the third trimester.

**Fig. 18:**
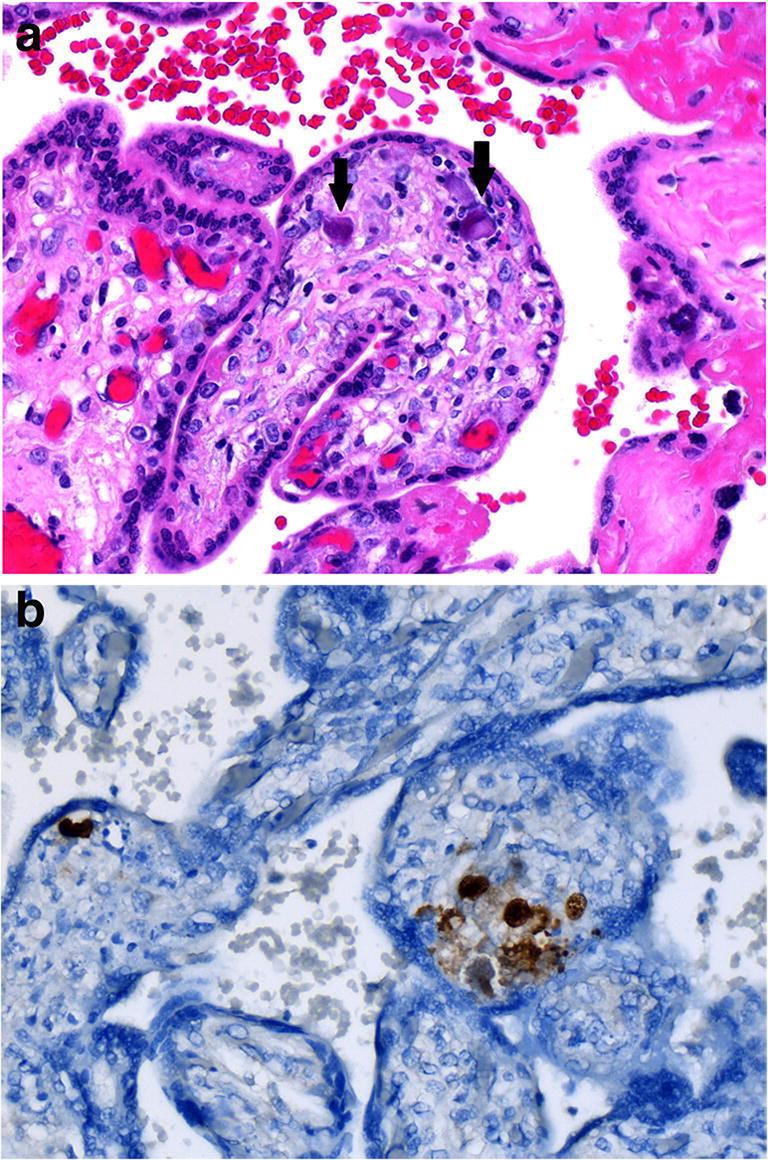
Cytomegalovirus infection in a placenta delivered at 27 weeks. The infant showed asymmetrical IUGR (456 g). Maternal serum detected IgM and IgG for CMV. The placental disc weighed 127 g. Coiling index 1 coil/5 cm. **a** Occasional intranuclear inclusions (black arrows) in endothelial cells of villous vessels (H&E, Original magnification ×400) **b** Inclusions show immunoreactivity for CMV (CMV imunostain, Dako, mouse monoclonal, 1/100 dilution, Original magnification ×400)

The chronic inflammatory infiltrates of infectious chronic villitis may include plasma cells, which are an important clue to the evaluating pathologist that the chronic villitis is infectious in origin. The TORCH viruses, such as cytomegalovirus, are most commonly a cause of chronic villitis (Fig. 18a and b). Correlation with microbiological culture remains important [9].

### Chronic (histiocytic) intervillositis of unknown etiology (massive chronic intervillositis)

**Fig. 19:**
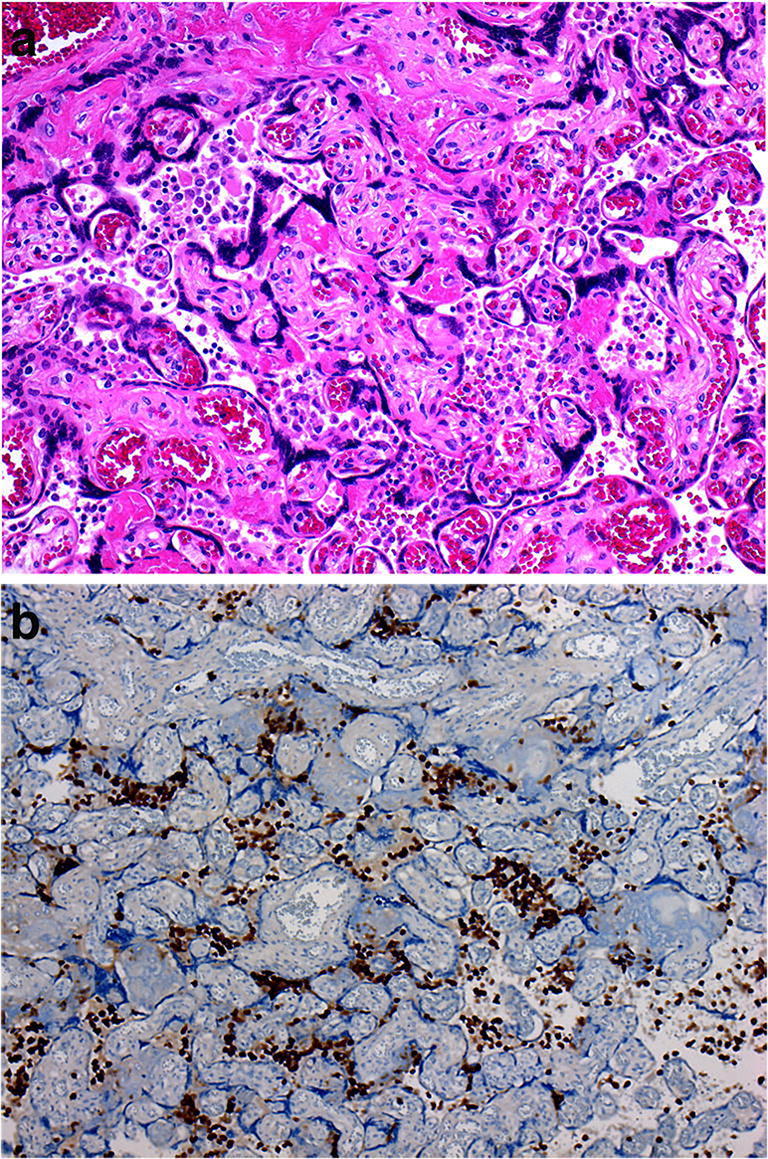
Diffuse chronic intervillositis in a placenta delivered at 37 weeks. The infant weighed 2.74 kg. Maternal placental ALKP 4082 U/L (NR 20–110 U/L). The placental disc weighed 380 g. Coiling index 1 coil/5 cm. **a** A diffuse infiltrate of mononuclear cells (mainly histiocytes and with some lymphocytes) in the intervillous place associated with some perivillous fibrin deposition (H&E, Original magnification ×200). **b** macrophages showing immunoreactivity for CD68 (CD68 immunostain, Cell Marque, mouse monoclonal, 1/600 dilution. Original magnification ×200)

Chronic intervillositis (CIUE) can be due to non-infective or infective causes. The infective cause is Malaria while the non-infective form is thought to be alloimmune. It is characterized by a diffuse infiltrate of mononuclear cells (histiocytes and lymphocytes) of maternal origin into the intervillous place that may be associated with some perivillous fibrin deposition (Fig. 19a and b). CIUE has a 4–100% chance of recurrence in a subsequent pregnancy and is frequently associated with fetal growth restriction, poor Apgar scores and fetal death [68]. CIUE can be a cause of first and second trimester loss with few pregnancies reaching term gestation. Macroscopically, the placental hypoplasia is common although no obvious macroscopic lesions are seen.

### Massive perivillous fibrinoid deposition and maternal floor infarction

**Fig. 20:**
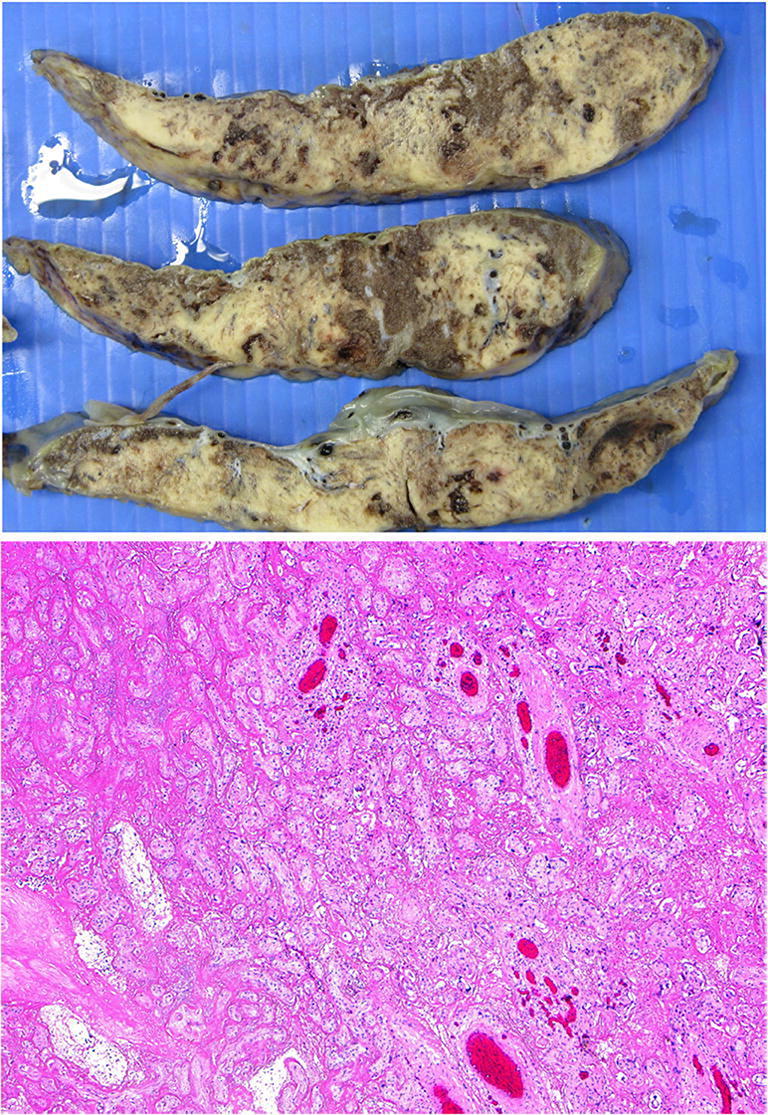
Massive perivillous fibrinoid deposition in a placenta delivered at 36 + 3 weeks. The infant weighed 2.0 kg. The placental disc weighed 317 g. Coiling index 1 coil/5 cm. **a** A diffuse excessive deposition of fibrinoid in the placental parenchyma. **b** Perivillous fibrin deposition resulting in obliteration of the intervillous space and secondary ischemic change of the villi (H&E, Original magnification ×50)

Massive perivillous fibrinoid deposition (MPFD) and maternal floor infarction (MFI) are related entities characterized by excessive deposition of fibrinoid in the placental parenchyma [9] (Fig. 20a and b). The pregnant patient with MPFD/MFI is typically clinically normal; a drop-off of fetal growth or decreased fetal movements in the late second or third trimesters may be the only indication of underlying pathology [9]. Elevation in maternal serum α-fetoprotein may be detected from the second trimester, with significant elevation in major basic protein levels in some patients [9]. In MPFD, the pattern of fibrinoid deposition is diffuse, while in MFI, the fibrinoid material is laid down along the maternal floor of the placenta [9]. The cause remains unknown, but suggested etiologies include autoimmune disease, activated protein C resistance, latent herpes infection and a toxic insult mediated by pregnancy -associated major basic protein ‘S ‘[69]. The process may represent a final common pathway from a variety of chorionic villus injuries in association with stasis of the intervillous circulation [70]. The clinical importance of these lesions relates to the associated poor outcome for the fetus (such as growth restriction, poor Apgar scores or fetal death usually in the third trimester) and for the risk of recurrence in future pregnancies of at least 20% [9].

### Metabolic storage diseases

**Fig. 21:**
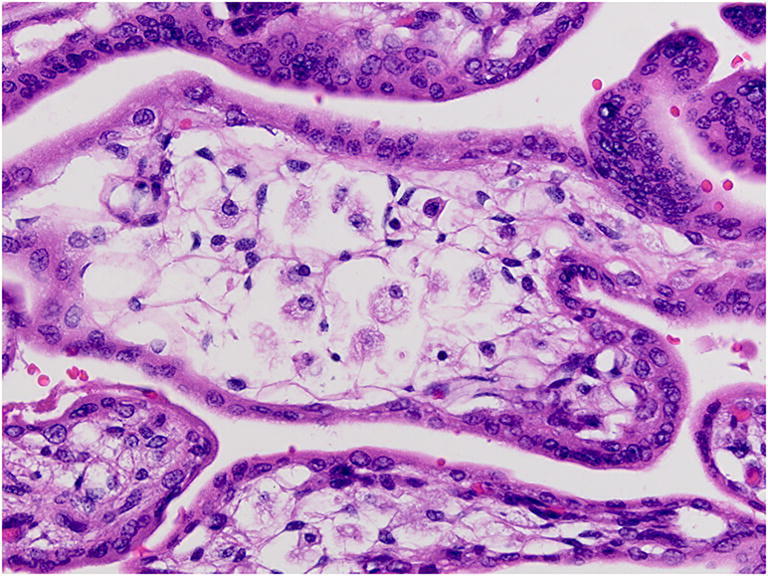
Mucopolysaccharidosis type IV in a placenta delivered at 17 weeks. The placental disc weighed 93 g. Coiling index 1 coil/5 cm. Granular vacuolated Hofbauer cells are seen in the villous stroma (H&E, Original magnification ×400)

Unsuspected fetal storage disorder may initially be diagnosed by routine placental examination of an initially normal-appearing neonate born to a previously unaffected family. Placental findings depend on the cause, but typically for lysosomal storage disease vacuolization of the stromal Hofbauer cells and or the syncytiotrophoblast or intermediate trophoblast may be seen (Fig. 21, courtesy of Dr. Lavinia Hallam, ACT Pathology) [71]. A review of the subsequent enzyme analysis is then required for a definitive diagnosis.

### Tumors in the placenta

**Fig. 22:**
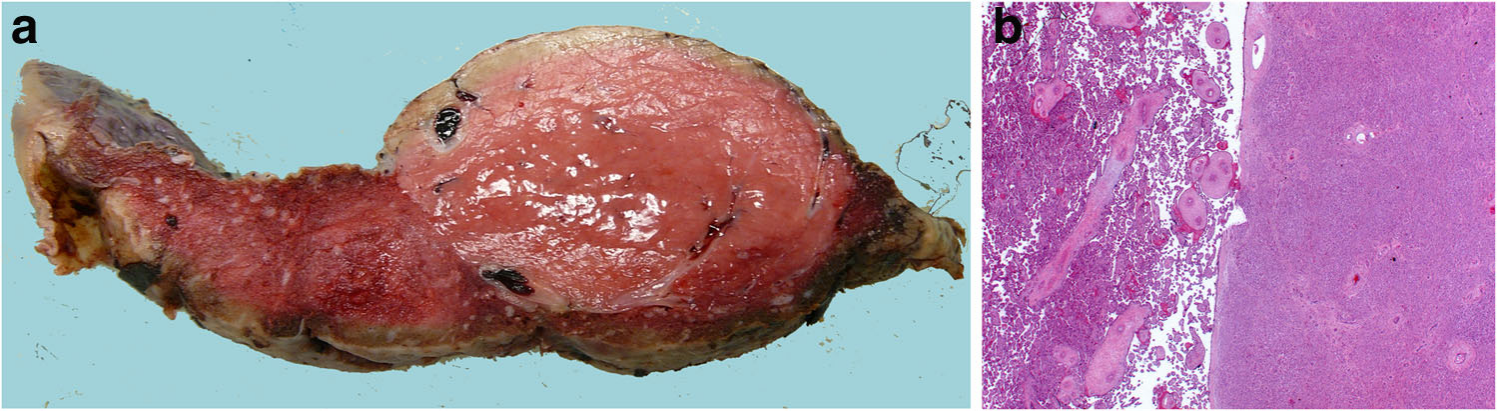
Chorangioma in a placenta delivered at 32 weeks. The placental disc weighed 450 g. Coiling index 1 coil/5 cm. **a** Well defined fleshy pink tumor 65 mm across. **b** well demarcated vascular tumor composed of capillary sized blood vessels (H&E, Original magnification ×12.5)

**Fig. 23:**
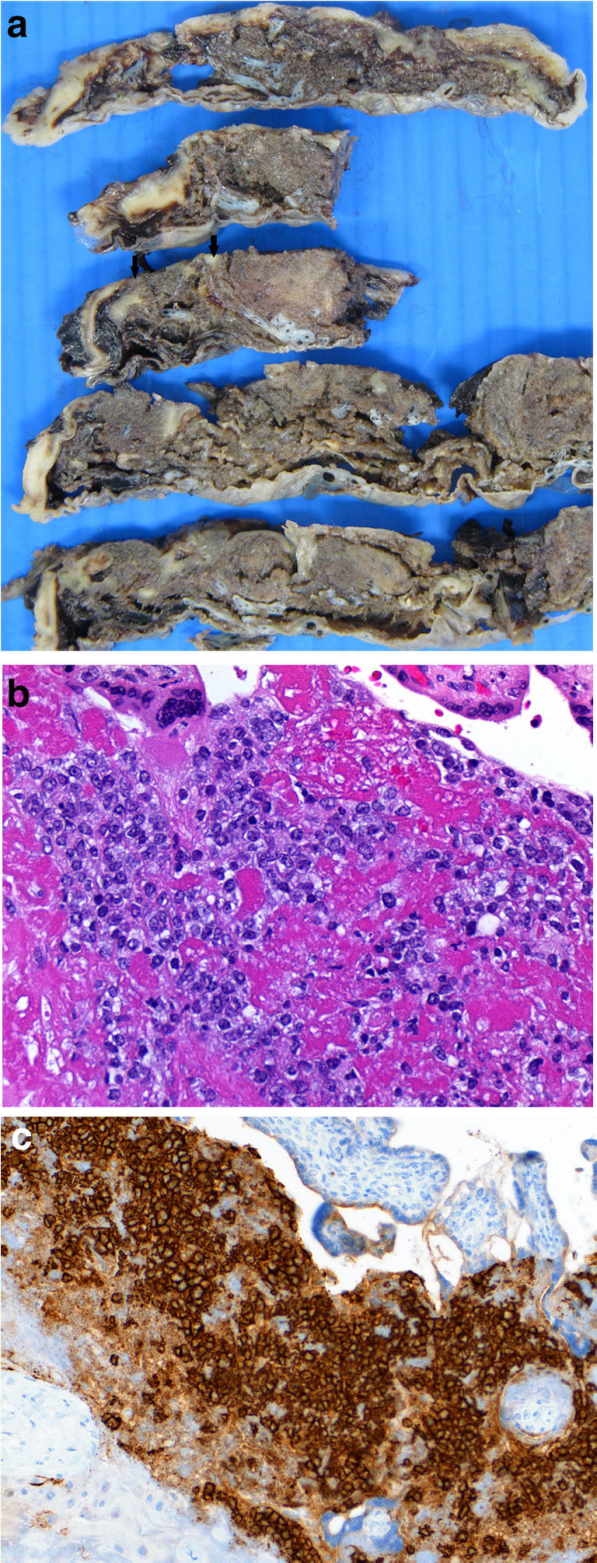
Diffuse large B cell lymphoma in a placenta delivered at 32 weeks. The placental disc weighed 450 g. Coiling index 1 coil/5 cm. **a** Ill-defined cream nodules less than 15 mm across on the basal plate aspect of the disc of varying size. **b** Infiltrate of large atypical cells within fibrin (H&E, Original magnification ×400) **c** (CD 20 [L26] immunostain, Ventana, mouse monoclonal, 1/100 dilution, Original magnification ×200)

**Fig. 24:**
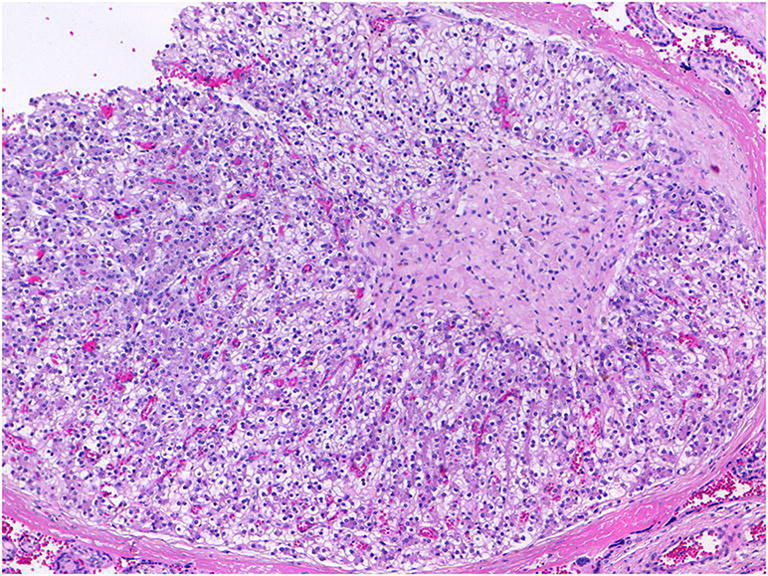
Adrenocortical rest in a placenta delivered at 40 weeks. The placental disc weighed 456 g. Coiling index 1 coil/5 cm. (H&E, Original magnification ×100)

The most common tumor of the placenta is a chorangioma which unless it involves around 30% of the placental disc area is of no clinical significance (Fig. 22a and b). While uncommon, placental examination may provide the diagnosis for an unexpected sudden maternal death through the detection of unexpected malignancy such as adenocarcinoma, lymphoma or melanoma. Most common metastatic maternal tumors are melanoma (30%) [72], breast carcinoma (14%), leukemia and lymphoma (15%), small cell carcinoma of lung [73] and gastric carcinoma [74]. Often the macroscopic assessment is unremarkable but microscopically malignant cells are seen often towards the basal plate (Fig. 23a–c). Occasional incidental findings include nodules of adrenocortical (Fig. 24) or liver tissue in the placenta which are of no clinical significance.

### Placenta accreta spectrum

**Fig. 25:**
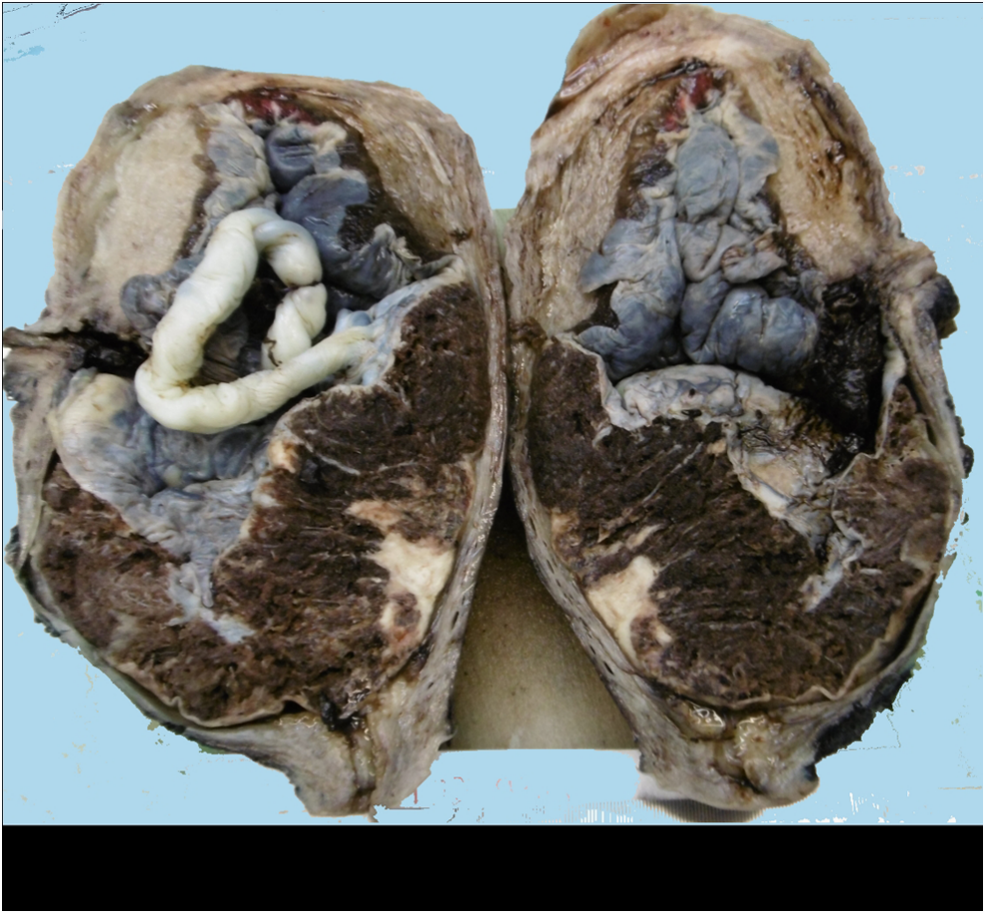
Placenta accreta delivered at 34 weeks. The placenta was morbidly adherent to the uterine cavity with markedly thinned retroplacental myometrial thickness. No myometrial invasion was identified histologically

Placenta accreta spectrum (PAS) is a general term used to describe abnormal trophoblast invasion into the myometrium of the uterine wall and includes placental accreta, increta and percreta. Macroscopically the placenta is morbidly adherent to the uterus (Fig. 25). Placenta accreta (anchoring placental villi attach to the myometrium rather than decidua) accounts for around 79% of cases, while placenta increta (anchoring placental villi penetrate into the myometrium) accounts for 14% and placenta percreta (anchoring placental villi penetrate through the myometrium to the uterine serosa or adjacent organs) for 7% of cases [75]. The first clinical manifestation of PAS is usually profuse, life-threatening hemorrhage that occurs at the time of attempted manual placental separation. Histologically, it can often be difficult to confirm an antenatal diagnosis of PAS without the uterus but careful macroscopic assessment of the maternal surface of the placenta may detect defects in the surface or adherent strands of muscle.

## Timing of placental pathology

It is often impossible to provide answers accurate to days or hours in placental pathology. Instead, it is often helpful to broadly distinguish between placental pathologies resulting in acute or chronic in utero compromise (Table 4) as a framework for determining the timing of the fetal insult [9, , 76]. Multiple placental lesions are often present and may be of different duration. Details of the timing of some placental lesions has been provided under specific entities above. By the timing of all lesions found, a sequence of events can often be reconstructed in the development of an adverse event [, 35]. Certain placental pathologies are more likely to result in stillbirth at particular gestations as described above, They summarized in Table 5.

**Table 4:** Placental findings indicating acute and chronic in utero compromise [9]

Placental findings indicating ***acute*** in utero compromise Normal placental weight or weight appropriate for fetal weight Acute villous oedema Fresh intravillous haemorrhage Acute retroplacental haemorrhage Acute meconium staining	
Placental findings indicating ***chronic*** in utero compromise (more than 48-72 h) Abnormal placental weight in relation to fetal weight Chorangiosis Fetal normoblastaemia Meconium-associated myonecrosis of cord vessel(s) Acute or necrotising funisitis High grade chronic villitis Chronic histiocytic intervillositis Maternal floor infarction/massive perivillous fibrinoid deposition Amnion nodosum Delayed villous maturation Maternal vascular malperfusion (including significant placental infarction, decidual arteriopathy) Fetal vascular malperfusion (chronic fetal vascular obstruction/fetal thrombotic Vasculopathy)

**Table 5:** Conditions that affect the placenta and can cause intrauterine death at various gestational ages

Disease	First trimester	Second trimester	Third trimester
Fetal vascular malperfusion		**+**	**+++**
Maternal vascular malperfusion		**++**	**+++**
Placental abruption		**+**	**+++**
Intervillous thrombus			**++**
Meconium staining			**+++**
Amniotic fluid infection with histological maternal± fetal inflammatory response		**+++**	**++**
Chronic intervillositis of unknown etiology (CIUE)	**+++**	**++**	**+**
Chronic villitis of unknown etiology (VUE)		**+**	**+++**
Massive perivillous fibrin deposition		**+**	**+++**

On occasions the forensic pathologist is asked to determine the timing of a fetal death in cases of home delivery of a stillborn versus a neonaticide, or allegations of medical negligence. Stillbirth results in involutional changes in the placenta that are well described [35] and recently well summarized by Boyd [77].

**Fig. 26:**
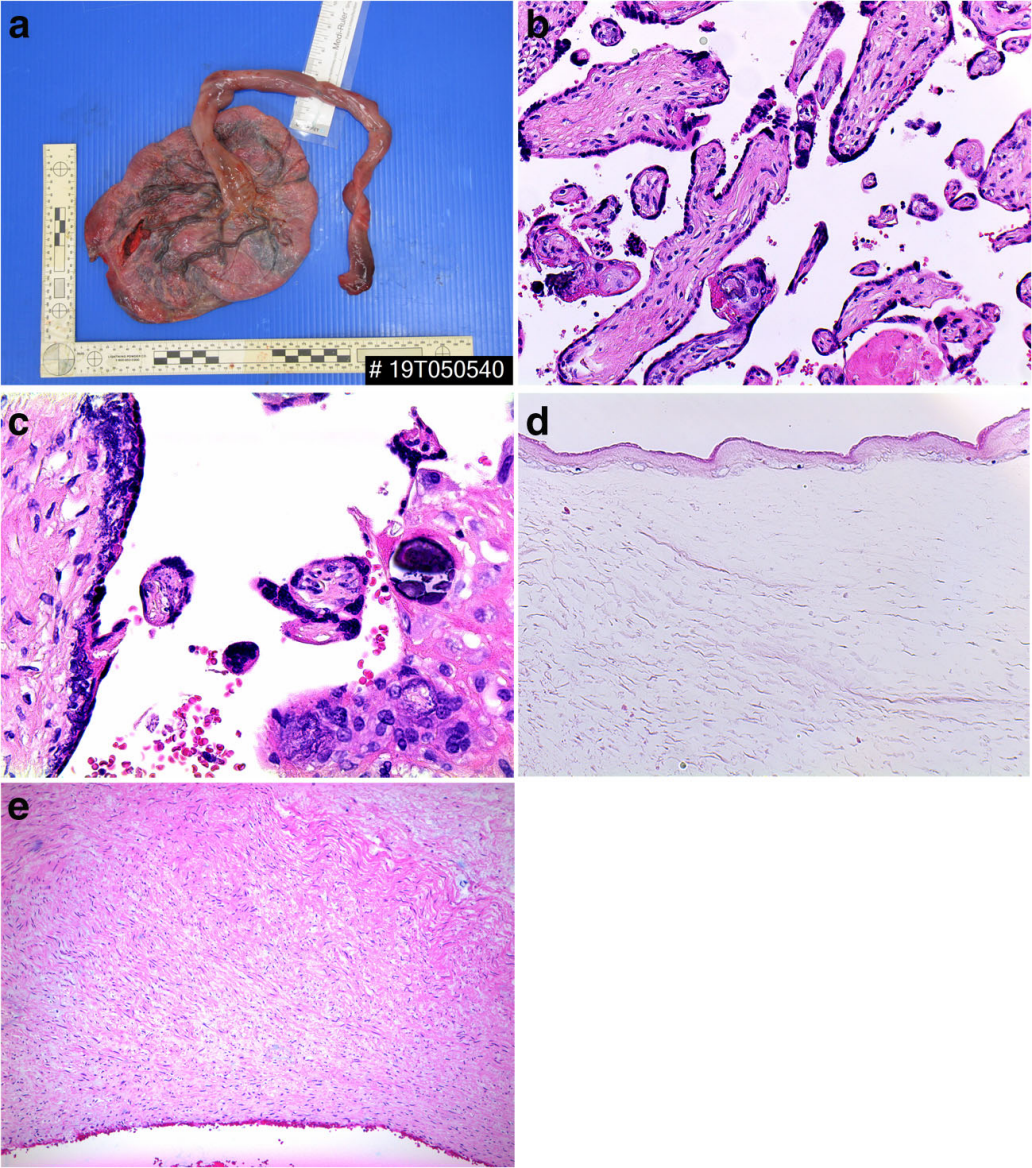
Fetal death in utero at 36 weeks. The placental disc weighed 319 g. The fetus showed grade II maceration and weighed 2332 g (normal range at 36 weeks = 2465 ± 294 g). Coiling index 1 coil/5 cm **a** Discolouration of the umbilical cord due to hemolysis. **b** Involution of fetal vessels and increased syncytial knot formation (H&E, Original magnification ×200). **c** Mineralization of the basement membrane of trophoblasts lining villi (H&E, Original magnification ×400) **d** Degenerate change of the amniotic epithelium of the extraplacental membranes (H&E, Original magnification ×400). **e** Degenerate change of the myocytes within vessel walls in the umbilical vein (H&E, Original magnification ×200)

**Fig. 27:**
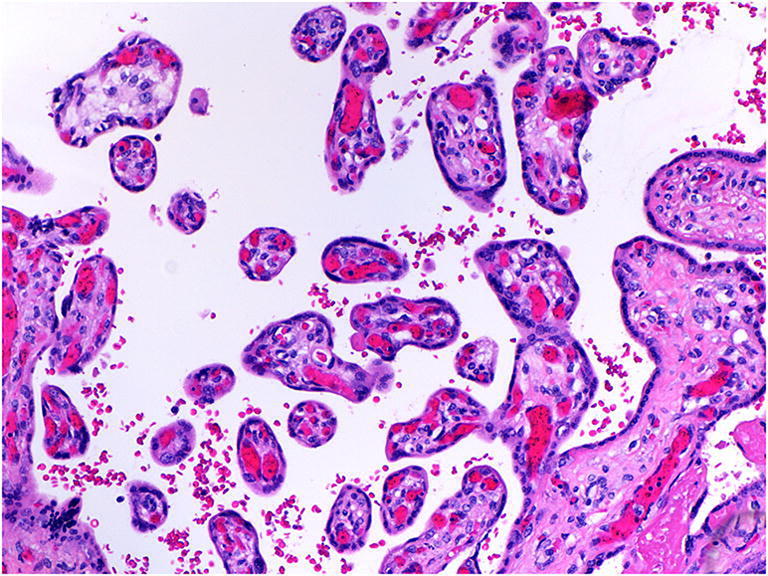
Fetal death in utero at 36 weeks. The placental disc weighed 317 g. The fetus showed grade 1 maceration and weighed 2000 g. Coiling index 1 coil/5 cm. Note the intravascular karyorrhexis seen at least 6 h after fetal death in utero membranes. Also present some nucleated red blood cells (H&E, Original magnification ×200)

Macroscopically for example, following intrauterine demise, one sees red-brown discoloration of the umbilical cord after 48 h due to hemolysis (Fig. 26a). Changes related to cessation of fetal blood flow identical to those seen as a result of fetal vascular malperfusion described above evolve over time but the features are global rather than regional. There is also altered maternal perfusion that results increased perivillous fibrin deposition and changes that overlaps with maternal vascular malperfusion such as increased syncytial knot formation (Fig. 26b). Mineralization of the basement membrane is due to the cessation of exchange of nutrient and waste (Fig. 26c). In addition, degenerate changes of the amniotic epithelium occur (Fig. 26d) and involutional changes of the vessels are seen in the umbilical cord with lifting of the endothelium and degenerative change of the myocyte indicative of maceration (Fig. 26e). The myocyte changes should not be confused with neutrophils. Placental changes with more recent fetal demise are more subtle. After 6 h one may see just intravascular karyorrhexis in fetal vessels (Fig. 27).

## Conclusion

The forensic pathologist examining a placenta is in the unique position of being able to assess possible traumatic lesions, in addition to being able to diagnose diseases of the mother and fetal/neonate. Information obtained through pathological examination of the placenta may be useful in subsequent obstetric care of the mother and in explaining past obstetrical history. It may also provide answers to unexpected fetal/ neonatal death. When there has been an adverse outcome to a pregnancy placental pathology may also be of considerable utility in informing parties involved in litigation.

The interpretation of placental findings in the forensic setting should not be divorced from the clinical details of the pregnancy, mother, and fetus/neonate. Consultation with a surgical pathologist with expertise in placenta examination is recommended as they often have access to, and expertise in interpretation of, ancillary tests not routinely used by forensic pathologists. Placental findings are usually context dependent, and determination of their significance often requires an appreciation and critical analysis of the entire clinicopathologic setting.

### Key points

1. Examination of the placenta may provide an explanation for fetal, neonatal or maternal demise.
2. In view of its key role as an interface organ evaluation of the placenta should be a mandatory part of all perinatal or maternal autopsies.
3. As published protocols for placental examination are not always followed this review details practical guidelines for this assessment.
4. In addition, involvement of local perinatal pathology services may provide additional expertise.
