# Correction to: A practical guide to placental examination for forensic pathologists

**DOI:** 10.1007/s12024-021-00441-6

**Published:** 2021-11-11

**Authors:** Namita Mittal, Roger W. Byard, Jane E. Dahlstrom

**Affiliations:** 1 Canberra Health, Services, ACT 2606, PO Box 11, Woden, Australia; 2 Faculty of Medicine, University of Adelaide, Adelaide, 5005 Australia; 3 Forensic Science SA, GPO, Box 2790, Adelaide, 5001 Australia; 4 College of Health and Medicine, Australian National University Medical School, ACT 2601, Canberra, Australia

## Correction to: Forensic Sci Med Pathol (2020) 16:295–312 https://doi.org/10.1007/s12024-019–00214-2

The article **A practical guide to placental examination for forensic pathologists** was originally published electronically on the publisher’s internet portal on 24 December 2019 without open access. After publication in volume [16], issue [2], pages [295–312] the author decided to opt for Open Choice and to make the article an Open Access publication. Therefore, the copyright of the article has been changed to © The Author(s) 2021 and the article is forthwith distributed under a Creative Commons Attribution 4.0 International License (https://creativecommons.org/licenses/by/4.0/), which permits use, sharing, adaptation, distribution and reproduction in any medium or format, as long as you give appropriate credit to the original author(s) and the source, provide a link to the Creative Commons licence, and indicate if changes were made.

The original article has been corrected.

